# Ultra‐Tough Copper–Copper Bonding by Nano‐Oxide‐Dispersed Copper Nanomembranes

**DOI:** 10.1002/advs.202408302

**Published:** 2025-02-14

**Authors:** Yun Teng, Wenqing Zhu, Qing Wang, Zhibo Zhang, Hang Wang, Baisong Guo, Ziyin Yang, Hao Gong, Chuan He, Boxi Qu, Shien‐Ping Feng, Yong Yang

**Affiliations:** ^1^ Department of Mechanical Engineering City University of Hong Kong Tat Chee Avenue, Kowloon Tong Kowloon Hong Kong 999077 China; ^2^ State Key Laboratory for Turbulence and Complex System Department of Mechanics and Engineering Science College of Engineering Peking University Beijing 100871 China; ^3^ Laboratory for Microstructures Institute of Materials Shanghai University Shanghai 200444 China; ^4^ Institute of Advanced Wear & Corrosion Resistant and Functional Materials Jinan University Guangzhou Guangdong 510630 China; ^5^ Department of Systems Engineering City University of Hong Kong Tat Chee Avenue, Kowloon Tong Kowloon Hong Kong 999077 China; ^6^ Nano and Advanced Materials Institute Science Park West Avenue Hong Kong Science Park Hong Kong 999077 China; ^7^ Department of Materials Science and Engineering City University of Hong Kong Tat Chee Avenue, Kowloon Tong Kowloon Hong Kong 999077 China

**Keywords:** copper nanomembranes, copper oxides, interfacial toughness, metal–metal bonding, strength‐ductility trade‐off

## Abstract

Metal–metal bonding has played a pivotal role in advancing human technologies across various industrial sectors. As devices continue to miniaturize, there is an increasing need for efficient bonding techniques capable of achieving metal–metal bonds at smaller length scales. In this study, a facile but effective bonding technique is developed that enables the bonding of randomly oriented copper with copper nanomembranes under low temperatures and pressures. The fabricated copper nanomembranes, with a thickness of ≈50 nm and a width of 1 cm or above, exhibit a unique heterogeneous nanostructure, comprising copper nanocrystals along with nano‐copper‐oxide dispersions. Consequently, these copper nanomembranes display exceptional mechanical properties, including an ultra‐low elastic modulus of ≈35 GPa, a remarkable yield strength of ≈1 GPa, and excellent ductility of ≈40%, overcoming the conventional strength‐ductility trade‐off observed in various copper alloys. Most importantly, these ultra‐soft copper nanomembranes serve as metallic “glues”, promoting grain growth across the bonding interface between randomly oriented copper surfaces. This process leads to an average interfacial shear strength of up to 73 MPa at room temperature, representing an approximate 35 times increase in bonding strength compared to direct copper–copper bonding achieved under identical temperature and pressure conditions.

## Graphical abstract

Conventional copper–copper bonding methods are typically costly and environmentally detrimental due to the use of various chemicals. In this study, a copper nanomembrane (NM) bonding approach is developed specifically designed for copper–copper bonding. This approach is marked by the large‐scale fabrication of flexible yet strong copper NMs using the polymer surface buckling enabled exfoliation method. Notably, this nanomembrane bonding approach is characterized by its cost‐effectiveness, environmental friendliness, and remarkable bonding strength.

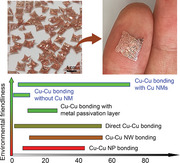

## 1 Introduction

Metal–metal bonding plays a critical role in various fields, including aerospace engineering,^[^ ^1^ ^]^ automobile manufacturing,^[^ ^2^ ^]^ semiconductor industry,^[^ ^3^ ^]^ and micro‐ and nano‐electronics.^[^ ^4^ ^]^ In the advanced packaging industry for next‐generation nano‐electronics,^[^ ^5^ ^]^ there is a pressing need to develop efficient yet easy‐to‐implement metal–metalmetal–metal bonding techniques at small length scales, particularly for copper–copper (Cu) bonding in 2.5D or 3D packaging.^[^ ^6^ ^]^ This demand arises as devices continue to miniaturize, aiming to surpass the performance limits imposed by empirical laws like Moore's law.^[^ ^7^ ^]^ To date, multiple methods have been developed for metal–metalmetal–metal bonding, broadly categorized into three types, namely, soldering/brazing, fusion welding, and solid‐state bonding.^[^ ^4^ ^]^ Soldering and brazing involve introducing molten filler metals between base components, resulting in bonding through metallurgical reactions.^[^ ^8^ ^]^ However, conventional filler metals like Sn‐Pb solder alloys are not environmentally friendly^[^ ^9^ ^]^ and, more importantly, have sizes larger than a few micrometers,^[^ ^10^ ^]^ making them unsuitable for metal–metal bonding at submicron scales. In comparison, fusion welding entails localized melting of base materials at high temperatures, requiring substantial energy inputs.^[^ ^11^ ^]^ Consequently, fusion welding is unsuitable for joining heat‐sensitive substrates, such as those with low transition temperatures like ferroelectric materials,^[^ ^12^ ^]^ as well as flexible electronics or organic light‐emitting devices.^[^ ^13^ ^]^ Similarly, solid‐state bonding, which typically involves significant plastic deformation in substrates at elevated temperatures, faces limitations when it comes to metal–metalmetal–metal bonding at the micro‐ or nano‐scale^[^ ^9^, ^14^ ^]^ due to stringent requirements on substrate materials (e.g., excellent plasticity, superior thermal stability, and considerable interdiffusion ability).

In recent years, there has been a growing interest in the development of bonding techniques utilizing nanomaterials. Nanomaterials offer several advantages over conventional materials, including their low melting temperatures and high percentages of surface atoms,^[^ ^15^ ^]^ which enable easy bonding between them.^[^ ^16^ ^]^ Moreover, nanomaterials can serve as effective fillers or interlayers to facilitate bonding between large‐sized metals with minimum energy inputs.^[^ ^16^ ^]^ For instance, Garnett et al.,^[^ ^17^ ^]^ successfully bonded Silver nanowires (30–80 nm in diameter) with a broadband tungsten–halogen lamp at a power density of 30 W cm^−2^ for 10–120 s, creating interconnected networks on a large scale at room temperature. Lu et al.,^[^ ^18^ ^]^ demonstrated the cold‐welding of single crystalline gold nanowires (3–10 nm in diameter) with identical crystal orientations, achieved within seconds through mechanical contact under relatively low applied pressures. Araullo–Peters et al.,^[^ ^19^ ^]^ developed AgCu/AlN multilayers, ranging in thickness from 4 to 10 nm, to achieve Cu─Cu bonding under 300 °C. While these nano‐bonding methods have shown promising results, they possess certain limitations. Some are restricted in terms of bonding size, such as the cold welding of Au nanowires.^[^ ^18^ ^]^ Others require complex and costly thermo‐chemical processes, such as formic acid pretreatment for deoxidation.^[^ ^20^ ^]^ Consequently, there is an urgent need for the further development of efficient nano‐bonding techniques that are both cost‐effective and practical.

Inspired by prior research^[^ ^6^ ^]^ and recent advancements in 2.5D and 3D packaging in the semiconductor industry,^[^ ^21^ ^]^ here we develop a novel Cu─Cu bonding method utilizing freestanding, ultra‐soft Cu nanomembranes (NMs) fabricated via the polymer surface buckling‐enabled exfoliation (PSBEE) technique.^[^ ^22^ ^]^ Our NM bonding technique exhibits notable advantages over traditional nano‐bonding methods, characterized by its environmental friendliness, cost‐effectiveness, and remarkable efficiency. Specifically, it achieves a significant 35 fold increase in shear strength for Cu─Cu bonding compared to direct Cu─Cu bonding achieved under identical temperature and pressure conditions. Notably, our approach overcomes the limitations observed in conventional Cu─Cu bonding techniques,^[^ ^23^ ^]^ which are often impeded by surface oxides^[^ ^24^ ^]^ and/or random crystalline orientation of Cu surfaces.^[^ ^25^ ^]^ In contrast, our NM bonding technique demonstrates successful bonding with an ultra‐high bonding strength in the presence of surface oxides, regardless of crystalline orientations.

## 2 Results and Discussion

### 2.1 Structural Characterization

**Figure 1:**
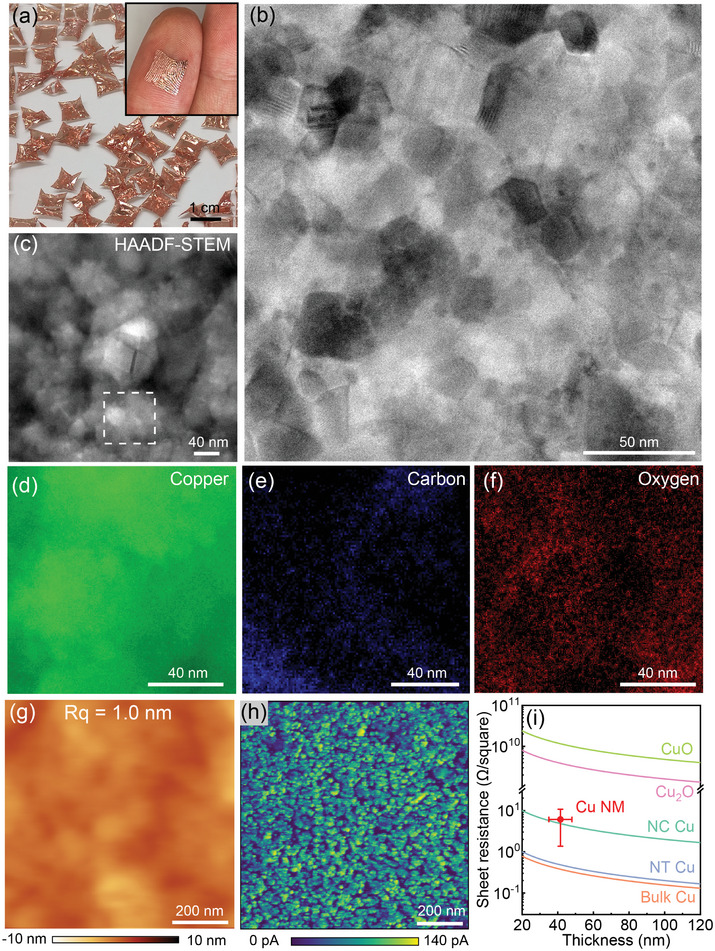
Structural characterization of the freestanding Cu NM fabricated using the PSBEE technique. a) Photograph showing the 48 nm‐thick Cu NMs peeled off from a PVA substrate and floating in water. The inset displays the Cu NM transferred onto a fingertip. b) Transmission electron microscopy (TEM) image showcasing the nanostructure of the Cu NM. c) High‐angle annular dark‐field scanning TEM (HAADF‐STEM) image with corresponding elemental mapping of d) Cu, e) C, and f) O. Elemental mapping region is highlighted by the dashed rectangle. g) Atomic force microscopy (AFM) scanning revealing the surface profile of the Cu NM. h) Electric current mapping of the Cu NM obtained through conductive AFM (C‐AFM) under an applied voltage of 5 mV. i) Comparison of the sheet resistance of the Cu NM with that of nanotwinned (NT) Cu,^[^ ^26^ ^]^ nanocrystalline (NC) Cu,^[^ ^26^ ^]^ bulk Cu,^[^ ^26^ ^]^ CuO^[^ ^27^ ^]^ and Cu_2_O.^[^ ^27^ ^]^

Following the PSBEE method,^[^ ^22^ ^]^ we successfully fabricated large‐area, freestanding ultrathin Cu NMs (**Figure** **1**a). For detailed fabrication procedures, please refer to Video S1 (Supporting Information) and Experimental Section. The thickness of these NMs ranged from 35 to 48 nm (Figure S1, Supporting Information), and their lateral size could extend to ≈1 cm or even larger. Remarkably, the inset in Figure 1a illustrates the exceptional conformability of these ultrathin Cu NMs, as they could make conformable contact with human fingers, revealing the fine textures of fingerprints. Transmission electron microscopy (TEM) images of a 48 nm‐thick Cu NM are presented in Figure 1b and Figure S2 (Supporting Information), demonstrating their nanocrystalline structure with an average grain size of 16 nm, along with various atomic‐scale defects such as twinning and stacking faults. In addition to Cu, scanning TEM energy‐dispersive X‐ray spectroscopy (STEM‐EDS) mapping (Figure 1c–f) confirmed the presence of C and O within these NMs. Selective area electron diffraction (SAED) patterns (Figure S2c, Supporting Information) and X‐ray photoelectron spectroscopy (XPS) spectra for Cu, C and O obtained at different etching times (Figure S3, Supporting Information) indicated that the Cu NMs mainly consisted of nanocrystalline Cu (90% in atomic percentage, 80% in volume fraction) and Cu (I) oxides (≈10% in atomic percentage, 20% in volume fraction), with a negligible fraction of C.

Atomic force microscopy (AFM) scans were conducted on the Cu NMs (Figure 1g,h and Experimental Section). Conductive AFM (C‐AFM) images revealed a heterogeneous distribution of local electric conductivity, with less conductive nano‐domains dispersed among highly conductive ones within the Cu NMs. The characteristic length of the heterogenous structure, determined from the height–height correlation function (Figure 1h), was ≈15 nm (Figure S4, Supporting Information), consistent with the average nanograin size measured from TEM images. The distribution of electric conductivity demonstrates that the Cu nano‐oxides are dispersed throughout the Cu NMs. We also measured the sheet resistance of the Cu NMs (Figure 1i) using the four‐point probe (FPP) method (see Experimental Section) and compared it with that of nanotwinned (NT),^[^ ^26^ ^]^ nanocrystalline (NC)^[^ ^26^ ^]^ and bulk Cu,^[^ ^26^ ^]^ CuO,^[^ ^27^ ^]^ and Cu_2_O.^[^ ^27^ ^]^ Notably, the electrical resistivity extracted for the Cu NMs was ≈25 µΩ·cm, similar to that of NC Cu^[^ ^26^ ^]^ (Table S1, Supporting Information). These findings suggest that the dispersed Cu nano‐oxides have minimum impact on the overall electrical conductivity of the Cu NMs.

### 2.2 Mechanical and Thermal Properties of Cu NMs

**Figure 2:**
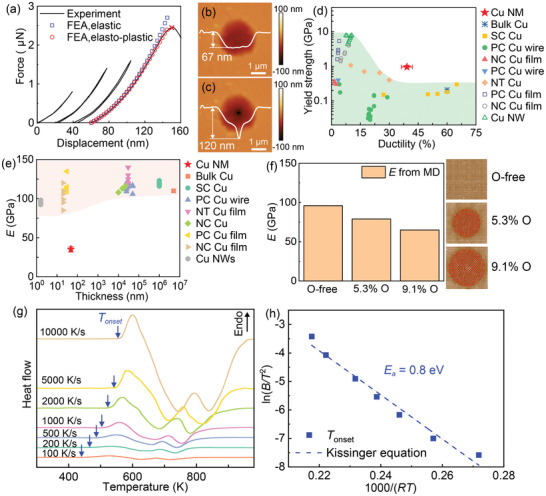
Mechanical and thermal properties of our Cu NMs. a) The experimental loading/unloading curves of a 48 nm‐thick Cu NM were obtained through AFM indentation with increasing load, alongside the finite element analysis (FEA) results. AFM scanning images of the suspended Cu NM b) before and c) after indentation. d) Comparison of the yield strength and ductility of the Cu NMs with other materials. e) Comparison of the elastic modulus of the Cu NM with other materials. f) Elastic modulus of pure Cu, Cu with 5.3% O and 9.1% O as determined from molecular dynamics (MD) simulations. g) FDSC heating curves of the Cu NMs with heating rates ranging from 10 000 to 100 K s^−1^. h) The activation energy of grain growth in the 48 nm‐thick Cu NM was determined by fitting the Kissinger equation to the experimental data.

To evaluate the mechanical properties of the Cu NMs, we performed AFM indentation tests on freestanding Cu NMs suspended over circular holes, following the well‐established circular drum indentation technique^[^ ^28^ ^]^ (Experimental Section). **Figure** **2**a illustrates the typical force‐displacement curves obtained from a 48 nm‐thick Cu NM, while Figure 2b,c presents AFM scanning images of the suspended Cu NM before and after indentation, revealing a ductile fracture mode similar to that observed in Au NMs.^[^ ^29^ ^]^ By fitting the experimental data to our finite element analysis (FEA) (Experimental Section) as described in previous studies,^[^ ^29^, ^30^ ^]^ we determined that these Cu NMs exhibited a yield strength of 950 ± 100 MPa and a ductility of 40 ± 3% (Figure 2d), surpassing various previously reported forms of Cu, including polycrystalline (PC) Cu,^[^ ^31^ ^]^ single crystalline (SC) Cu,^[^ ^32^ ^]^ NC Cu,^[^ ^33^ ^]^ NT Cu,^[^ ^26^ ^]^ bulk Cu,^[^ ^26^ ^]^ and Cu nanowires (NWs).^[^ ^34^ ^]^ Notably, the strong and ductile Cu NMs displayed a relatively low elastic modulus of *E* = 35 ± 3 GPa (Figure 2e), approximately one third of the bulk Cu,^[^ ^26^ ^]^ resulting in a superior elastic strain limit of 2.7 ± 0.3% (Figure S5, Supporting Information). Molecular dynamics (MD) simulations, as discussed later, revealed that even a small atomic fraction of Cu_2_O nano‐oxides (e.g., an atomic ratio of 5%–9% for O corresponding to a volume fraction of 20%–40% for Cu_2_O) can significantly diminish the elastic modulus of Cu (Figure 2f). Furthermore, we observed numerous defects such as twin boundaries and stacking faults were observed in our NMs (Figure S2d–g, Supporting Information), further diminishing their elastic modulus. Considering the collective effects of Cu_2_O and lattice defects, our MD simulations indicate that the elastic modulus extracted from unloading curves decreases with increasing O concentration, which could reach ≈60 GPa (Figure S6, Supporting Information). This trend aligns with our experimental observations, suggesting that the decreased modulus of Cu NMs is a result of Cu_2_O presence and abundant crystalline defects within them.

In addition to investigating the mechanical properties, we also explored the thermal stability of our Cu NMs via flash differential scanning calorimetry (FDSC) (see Experimental Section). The FDSC curves of the Cu NMs, obtained with heating rates ranging from 10 000 to 100 K s^−1^, revealed distinct exothermic peaks occurring between 600 and 750 K (Figure 2g). To gain insights into the underlying physical mechanisms driving these exothermic reactions, we conducted isothermal annealing of the Cu NMs for 30 s at 573 and 673 K (see Experimental Section). Upon TEM examination, we observed significant grain growth in the Cu NMs (Figure S7, Supporting Information), indicating that the exothermic peaks can be attributed to grain growth. By fitting the onset temperature of the exothermic reactions to the Kissinger equation, we determined an activation energy (*E_a_*) of 0.8 eV (Figure 2h), which closely matches the activation energy associated with grain growth in bulk NC Cu.^[^ ^35^ ^]^

### 2.3 Cu─Cu Bonding with Cu NMs

To initiate the Cu─Cu bonding process, we began by depositing a layer of Cu onto a Si wafer using magnetron sputtering. The deposited Cu could either be patterned using a shadow mask or left unpatterned as the bonding substrates (Figure S8, Supporting Information, and Experimental Section). The deposited Cu had a thickness of 3.5 mm, with a surface roughness of ≈5 nm (Figure S9a,b, Supporting Information) and an average grain size of ≈136 nm (Figure S10, Supporting Information). The elastic modulus of the ultrafine‐grained Cu was determined to be ≈101 GPa through nanoindentation, where the measured reduced modulus was fitted to King's model^[^ ^36^ ^]^ (see Figure S9c, Supporting Information, and Experimental Section). Subsequently, we conducted direct Cu─Cu bonding experiments without the application of Cu NMs. It is important to note that our bonding approach differed from previous methods^[^ ^37^ ^]^ which typically involved various surface treatments such as plasma and chemical mechanical planarization (CMP) of Cu surfaces prior to bonding (see Table S2, Supporting Information). In our case, we carried out the direct Cu─Cu bonding experiments without any surface treatment at different temperatures (e.g., 100, 200, and 300 °C) and for varying time periods (e.g., 3, 30, and 300 min). To ensure contact between the Cu surfaces, we applied a minimum force of 100 N throughout our bonding experiments using our bonding machine (ETOOL, ET‐1212‐300) (Figure S11, Supporting Information and Experimental Section). As shown in Figure S12 (Supporting Information), Cu─Cu bonding occurred when the temperature reached 200 °C with a bonding time of 300 min, or when the temperature reached 300 °C regardless of the bonding time. Subsequently, a selected number of Cu NMs were transferred onto the Cu surface (Video S2, Supporting Information). Due to their ultrathin thickness, the Cu NMs conformed closely to the Cu surface upon contact. For comparison with the direct Cu─Cu bonding results, we performed NM bonding at the temperature of 300 °C for varying times (3, 30, and 300 min) to facilitate the analysis.

**Figure 3:**
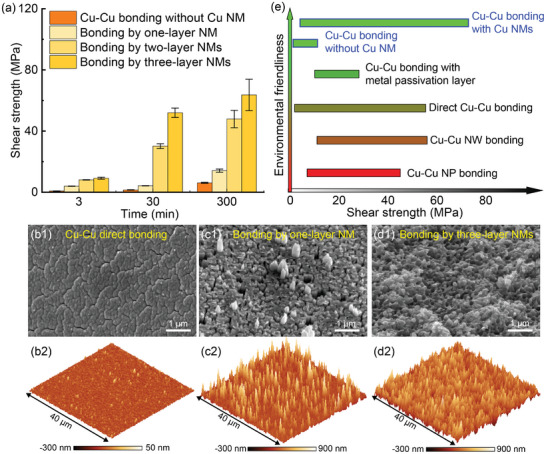
Tough, cost effective, and environmentally friendly Cu─Cu NM bonding. a) A comparison of measured shear strengths between Cu─Cu bonding with and without Cu NMs. (b1) SEM and (b2) AFM images of the fractured surface resulting from Cu─Cu bonding without Cu NMs at a bonding time of 300 min. (c1) SEM and (c2) AFM images of the fractured surface resulting from Cu─Cu bonding with one layer of Cu NM at a bonding time of 300 min. (d1) SEM and (d2) AFM images of the fractured surface resulting from Cu─Cu bonding with three layers of Cu NMs at a bonding time of 300 min. e) A comparison of our NM bonding technique with other Cu─Cu bonding techniques in terms of bonding strength and environmental friendless. All SEM images were taken at a tilt angle of 45° with respect to normal incidence.

Following the approach outlined in refs. [37, 38] we utilized the Nordson Dage 4000 Bond Tester to measure the average shear strengths of the bonded Cu─Cu surfaces, both with and without Cu NMs (see Videos S3 and S4, Supporting Information, and Experimental Section). To prevent potential brittle fracture in Si due to excessively strong Cu─Cu, we employed patterned Cu as the bonding medium. **Figure** **3**a illustrates the measured shear strengths for Cu─Cu bonding with and without Cu NMs. Evidently, with an increased number of transferred Cu NMs onto the Cu substrate, a notable improvement in bonding strength was observed, notably pronounced at bonding durations of 30 or 300 min. For instance, at a bonding time of 30 min, the average bonding strength was ≈1.5 MPa for direct Cu─Cu bonding. However, this value increased to 4 MPa with the addition of one layer of Cu NM and further rose to 52 MPa with three layers of Cu NMs, representing a remarkable 3500% increase. By extending the bonding time to 300 min with three layers of Cu NMs, the bonding strength could be further enhanced to 73 MPa. Notably, this value exceeded the reported Cu─Cu bonding strengths achieved under similar temperature and time conditions using various bonding techniques (Table S2, Supporting Information).

Subsequently, we conducted an analysis of the fractured surfaces following the debonding process between the bonded Cu─Cu surfaces. Notably, we observed a fascinating transition in the morphology of interfacial fracture from brittle to tough as the number of transferred Cu NMs increased, as depicted in Figure 3b–d and Figure S13 (Supporting Information). In the case of direct Cu─Cu bonding without Cu NMs (Figure 3b1,b2; Figure S13a–c, Supporting Information), the fracture surface exhibited a cleavage‐like morphology, consistent with weak interface bonding and brittle fracture. In contrast, Cu─Cu bonding with one layer of Cu NM displayed numerous protrusions on the fracture surface (Figure 3c1,c2; Figure S13d–f, Supporting Information). Moreover, Cu─Cu bonding with three layers of Cu NMs resulted in a rugged morphology with significant protrusions and dimples (Figure 3d1,d2; Figure S13g–l, Supporting Information). According to our optical microscopy findings, as shown in Figure S14 (Supporting Information), we observed a distinct smooth fracture zone at the periphery of the typical fracture regions characterized by dimples. According to refs. [39], such smooth fracture zones typically indicate the initiation of fractures, hence implying that the initial cracking likely originated from the interface between the Cu and the Cu NM. These rugged fracture surface morphologies indicate the presence of a tortuous cracking path along the bonded Cu─Cu surfaces, a characteristic typically associated with tough interfacial fracture.^[^ ^40^ ^]^

In addition to bonding strength, it is essential to consider the cost and environmental friendliness when developing Cu─Cu bonding techniques.^[^ ^41^ ^]^ These factors are closely related to the use of potentially harmful chemicals in the bonding process. In our study, we assessed the environmental impact and cost associated with various bonding techniques by examining the chemicals employed, such as NaOH, HCl, H_2_SO_4_, and toxic hydrazine monohydrate (please see Table S2, Supporting Information for details). Our Cu NM bonding method stands out in terms of cost, environmental friendliness, and ultra‐high bonding strength (Figure 3e). This is primarily due to the exclusive use of deionized (DI) water for producing Cu NMs, which significantly reduce the fabrication cost of nanomaterials. Furthermore, our method eliminates the need for surface treatments, further enhancing its cost‐effectiveness and environmental sustainability. Therefore, our Cu NM bonding technique offers a compelling advantage over other bonding techniques in terms of its low cost, environmental friendliness, and ability to achieve exceptionally high bonding strength (Figure 3e).

### 2.4 Toughening Mechanisms in NM Bonding

**Figure 4:**
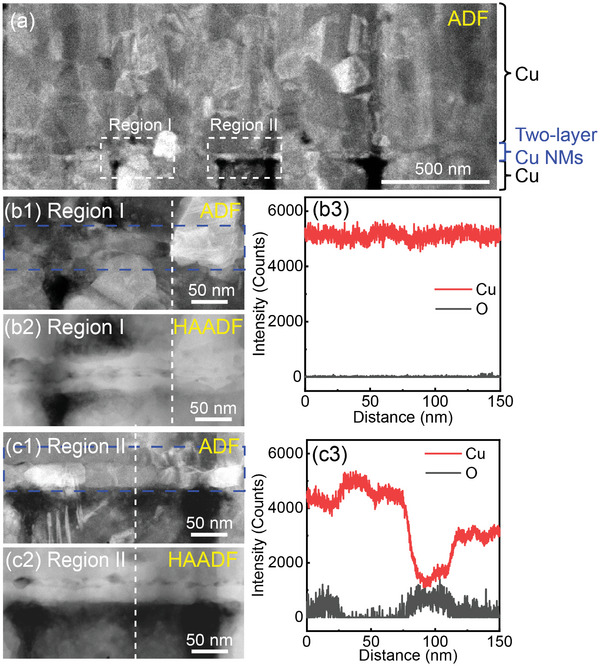
TEM Characterization of the bonding interface in NM bonding. (a) Annular dark‐field (ADF) TEM image of the bonding interface of Cu─Cu bonding with two layers of Cu NMs at the bonding time of 300 min. (b1) HAADF‐STEM and (b2) ADF TEM images of region I in (a). (b3) The elemental line scans along the dashed lines in (b1) and (b2). (c1) HAADF‐STEM and (c2) ADF TEM images of region II in (a). (c3) The elemental line scans along the dashed lines in (c1) and (c2). The two‐layer Cu NMs in (b1) and (c1) are highlighted by the dashed rectangles.

To gain insights into the toughening mechanisms of Cu─Cu bonding with Cu NMs, we employed the focused‐ion beam technique to perform a cross‐sectional cut of a bonded sample (see Experimental Section) and conducted TEM analysis of the bonding interface. TEM images in **Figure** **4**a depict the bonded Cu─Cu interface with two layers of Cu NMs. Notably, the presence of Cu NMs effectively sealed “voids” or “nano‐sized gaps” that are typically observed in direct Cu─Cu bonding (Figure S15a1,a2, Supporting Information). Additionally, we observed significant growth of nanocrystals across the bonded interface in regions without Cu oxides (e.g., Region I in Figure 4a). This growth of Cu nanocrystals spanning the two‐layer Cu NMs is supported by the HAADF‐STEM images and EDS analysis (Figure 4b1–b3). In contrast, regions containing substantial amounts of Cu oxides (region II in Figure 4a) impeded the growth of Cu nanocrystals most likely due to the presence of Cu oxides (Figure 4c1–c3). Remarkably, within these oxide‐rich regions, we observed cracking occurring within the Cu crystals in the neighborhood of Cu NMs, accompanied by crack‐tip blunting, signifying ductile fracture at the nano‐scale (Figure S15b1,b2, Supporting Information). Conversely, in cases of direct Cu─Cu bonding, cracking resulted in a sharp crack tip at the atomic scale, and fracture took place along the interface (Figure S15a2, Supporting Information). These TEM results provide compelling evidence of cleavage‐type interfacial fracture in direct Cu─Cu bonding, while Cu─Cu bonding with Cu NMs exhibits ductile fracture. The toughening mechanism inherent in Cu NM bonding is linked to a tortuous cracking pathway, as illustrated in Figures S13g–l and S16 (Supporting Information). This pathway is a consequence of interface heterogeneities arising from the substantial growth of Cu nanocrystals in regions devoid of nano‐oxides and the tough interfacial bonding in areas abundant with nano‐oxides. These observations align with the fracture surface morphologies presented in Figure 3b–d.

**Figure 5:**
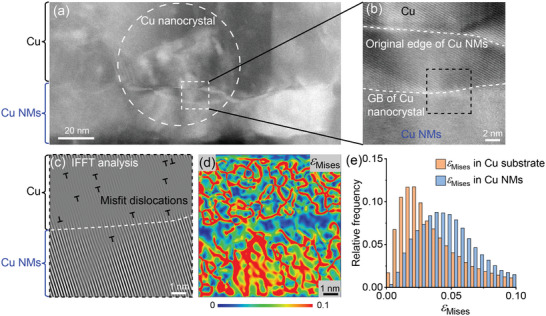
Characterization of elastic stress near the advancing edge of a growing nanocrystal within the Cu NM. a) ADF TEM image of the Cu nanocrystal that extended from the Cu substrate into Cu NMs (highlighted by the dashed cycle). b) ADF TEM image depicting the current grain boundary of the Cu nanocrystal. c) IFFT‐filtered HRTEM image of the region at the grain growth front, indicated by the black dashed rectangle in (b). d) Contour plot illustrating the distribution of von Mises strain ε_Mises_ in (c). e) Distribution analysis of von Mises strain ε_Mises_.

In order to gain a deeper understanding of how Cu grain growth is facilitated by Cu NMs, we conducted an examination of the leading edge of a Cu nanograin that originated from the Cu substrate and extended into the Cu NM, as depicted in **Figure** **5**a. The lattice misorientation at the grain growth front is highlighted in Figure 5b. By applying inverse Fast Fourier Transformation (IFFT) analysis to the HRTEM image (Figure 5c), we observed a significant presence of misfit dislocations in the region of the Cu substrate, whereas the Cu NMs displayed only a few dislocations. The lattice strain was calculated using geometric phase analysis (GPA)^[^ ^42^ ^]^ (Figure 5d,e; Figure S17, Supporting Information). Notably, there was a relatively large von Mises strain field in the Cu NMs compared to the Cu substrate, despite the former having fewer defects. This behavior indicates that the Cu NMs undergo elastic shearing during grain growth. Essentially, this elastic shearing within the Cu NMs can be explained by the elastic mismatch between the Cu NM (with *E* = 35 GPa) and the Cu substrate (with E = 101 GPa) when they are pressed together. A similar phenomenon was reported by Lu et al., where the application of stress to NC Cu, induced by cold rolling, promoted grain growth during an isothermal process.^[^ ^35^ ^]^ Moreover, our experiments revealed a low activation energy for grain growth within Cu NMs (Figure 2h). Consequently, once Cu nanocrystals crossed the bonding interface and entered the elastically stressed Cu NMs, they could easily propagate throughout the entire Cu NMs, as observed in Figure 4a.

### 2.5 Atomistic Mechanisms of NM Bonding

**Figure 6:**
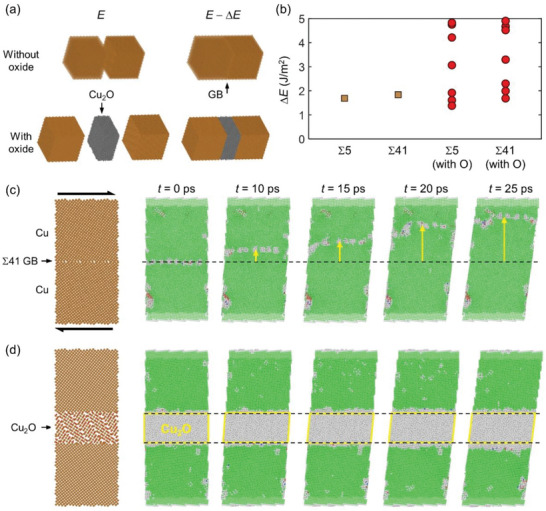
Molecular dynamics (MD) simulations of Cu‐Cu bonding with and without Cu NMs. a) Atomistic models before and after bonding are depicted, showcasing two different tilt GBs (Σ5[001](210) and Σ41[001](910)) for the bi‐crystal models. b) The energy difference per unit surface area after the occurrence of atomic bonding. Scatter points represent models with randomly oriented oxide interlayers. c,d) Snapshots of the bi‐crystal Cu model with Σ41[001](910) GB under simple shear, with and without an interlayer of cuprous oxide, respectively. Green, red, and blue spheres represent FCC, HCP, and BCC atoms, respectively, while gray color represents other types. The arrows in (c) indicate the grain growth direction.

To further understand the bonding mechanisms, we conducted MD simulations using bi‐crystal copper models with coincidence‐site‐lattice tilt GBs (see Experimental Section). Additionally, we investigated scenarios involving a Cu_2_O interlayer based on the experimental observations of dispersed Cu_2_O within the Cu NMs. Structural relaxation was performed both before and after bonding, as depicted in **Figure** **6**a. We calculated the thermodynamic driving force for bonding Δ*E* (i.e., the decrease of energy after bonding) in the bi‐crystal Cu and Cu_2_O||Cu models with different misorientations (Figure 6b). For the bi‐crystal Cu, we observed that Δ*E =* 1.9 J/m^2^ in the Σ41[001](910) model with a misorientation angle of 12.68°, which was higher than Δ*E =* 1.7 J/m^2^ in the Σ5[001](210) with a misorientation angle of 53.13°. This finding aligns with the commonly adopted strategy of metal–metal bonding, which involves minimizing misorientation to facilitate easier bonding.^[^ ^18^ ^]^ However, we also discovered that the driving forces for the Cu_2_O||Cu interface with oxide layers could be further increased to 1.5–5 J m^−2^. It is worth noting that the different data points correspond to Cu_2_O samples with randomly assigned orientations, resulting in misfit angles ranging from 14° to 57° between (100)_Cu_ and (100)_Cu2O_ faces.

In addition to thermodynamic considerations, we explored the temporal evolution of the bonded interfaces. We analyzed the GB migration kinetics responsible for grain growth across the interface under shear strain, as evidenced in our NMs through strain mapping (Figure 5d; Figure S17c,d, Supporting Information). The simulation results indicated that the GB mobility is dependent on the misorientation angle, with the GB migrating much faster in the Σ41[001](910)) model (Figure 6c) compared to the Σ5[001](210) model with a misorientation angle of 53.13° (Figure S18, Supporting Information). Conversely, the presence of an oxide interlayer completely suppressed interface kinetics (Figure 6d). Although Cu oxides impede the growth of Cu nanocrystals, they also play a positive effect on Cu NM bonding due to their strong driving force to bond with Cu nanocrystals. Importantly, our MD simulation results indicate significant grain growth in the Cu NM under shear strain, consistent with the enhanced GB migration observed in Figure 5.

**Figure 7:**
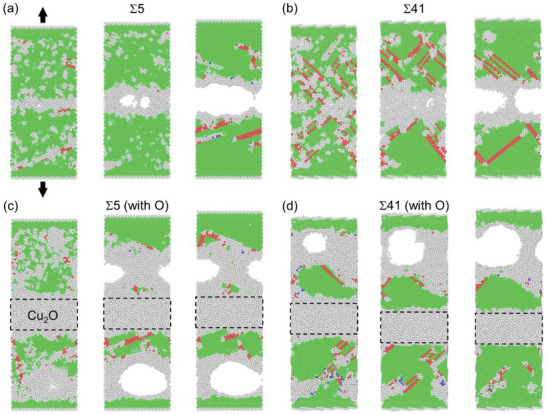
MD simulations of fracture in bonded Cu─Cu with and without cuprous oxide. Snapshots of fracture processes in bi‐crystal copper with Σ5[001](210) GB a) and Σ41[001](910) GB b), with an interlayer of cuprous oxide c,d) under uniaxial tensile strains of 17.5%, 20% and 22.5%. Green, red, and blue spheres represent FCC, HCP, and BCC atoms, respectively while gray color represents other types.

Subsequently, we investigated the interfacial fracture processes of the bonded models. Uniaxial tension was applied to the atomistic models with the loading direction perpendicular to the interface. In the bi‐crystal models without oxide, we observed damage initiation at the GBs, followed by the development of fully formed interfacial cracks (**Figure** **7**a,b), which is consistent with the fracture path observed in Cu─Cu direct bonding (Figure S15a1,a2, Supporting Information). These fracture processes were accompanied by the generation of stacking faults to dissipate the elastic energy. In contrast, interfaces in models with a Cu_2_O interlayer did not exhibit crack formation. Instead, void nucleation and growth occurred inside the copper nanograins at high strains, demonstrating a ductile fracture behavior (Figure 7c,d), which is in line with the experimental findings (Figure S15b1,b2, Supporting Information). This indicates that, in addition to growing Cu crystals, the presence of the nano‐dispersed oxide is also beneficial for the formation of a strong and ductile bonding interface in Cu NM bonding.

## 3 Conclusion

In summary, we have developed an NM bonding technique by harnessing the unique combination of structural, mechanical, and physical properties of Cu NMs fabricated through PSBEE. The presence of nano‐oxide dispersions in our Cu NMs imparts an exceptionally low elastic modulus, even when compared to monolithic Cu thin films of similar thickness. This remarkable softness of the Cu NMs leads to a significant elastic mismatch at the bonding interface when bonded to a Cu substrate, generating substantial shear stress within the Cu NMs. The fast atom diffusion kinetics observed in the Cu NMs during iso‐thermal annealing experiments, coupled with the local shear stressing due to elastic mismatch, facilitates grain growth across the Cu NMs. Although the nano‐oxide dispersions hinder grain growth within the Cu NMs, they exhibit strong bonding with Cu due to a high thermodynamic driving force. Our experiments and atomistic simulations reveal at the nano‐scale, the oxide‐Cu interface proves tougher than Cu, thereby preventing crack initiation at the bonded interface. The combination of substantial grain growth in Cu‐rich regions and a tough interface in nano‐oxide‐rich regions results in an ultra‐tough and efficient Cu NM enabled Cu─Cu bonding, regardless of Cu orientations. By increasing the number of Cu NMs from one to three, the average shear strength of the Cu‐NM‐enabled Cu─Cu bonding is enhanced by 35 times compared to that of Cu─Cu direct bonding achieved under identical experimental conditions. Importantly, the PSBEE method used for fabricating Cu NMs is environmentally friendly and cost effective,^[^ ^43^ ^]^ making it suitable for mass production. Thus, we anticipate that the NM bonding technique demonstrated here holds great potential for various applications, including the manufacturing of modern organic microelectronic/optoelectronic devices, nanoelectronics, and nanoelectromechanical systems.

## 4 Experimental Section

### Synthesis of Cu NMs and Cu Substrates

Cu NMs were fabricated with thicknesses ranging from 35 to 48 nm using the PSBEE method previously reported.^[^ ^22^, ^43^ ^]^ First, a layer of polyvinyl alcohol (PVA) was spin‐coated on the surface of a glass plate to obtain a hydrogel thin film and dehydrated it in a drying oven at 80 °C for 1h. Then, Cu was deposited onto the hydrogel thin film using electron beam evaporation (Junsun Tech LTD, EBS‐500F) at a high vacuum (with the pressure of 6 × 10^−4^ Pa). The Cu‐hydrogel‐glass system was immersed in deionized (DI) water. After a few minutes, the freestanding Cu NMs continuously delaminated from the hydrogel substrate (Video S1, Supporting Information). For the Cu substrates, they were fabricated by depositing Cu onto Si wafers using magnetron sputtering (Sky Technology Development LTD, JGP560) (Figure S8, Supporting Information). During the deposition process, the processing chamber's base pressure to 6 × 10^−4^ Pa was reduced and then refilled with 30 sccm Ar gas flow to maintain the working pressure at 1.3 Pa. A 50 nm‐thick Ti adhesion layer was first deposited on Si wafers, followed by sequential sputtering of a 3.5 µm‐thick Cu substrate with a size of 8 × 8 mm^2^, which served as the bottom wafer for Cu─Cu bonding. Additionally, a mask to produce patterned Cu substrates were also utilized, which were deposited on Si wafers with a size of 3 × 3 mm^2^.

### Structure and Electronic Properties Characterization of Cu NMs

Various techniques were employed to characterize the structure, chemical bonding, and electronic properties of Cu NMs. The Cu NMs were transferred onto TEM grids, Si wafers, or glass slices for analysis. The thickness and surface profile of the Cu NMs on Si wafers were examined using AFM (Oxford Instruments, MFP‐3D Infinity). Conventional field emission TEM (JEOL, JEM‐2100) operating at an acceleration voltage of 200 kV was used to acquire TEM images and SAED patterns of the Cu NMs on TEM grids. HAADF‐STEM, annular bright filed (ABF), DF imaging, and elemental mapping were performed using a Cs‐corrected thermal field emission TEM (JEM, ARM200F) at 300 kV. High resolution XPS was employed to study the chemical bonding in the Cu NM. The sheet resistance (*R_s_*) of the Cu NMs on glass slices was measured using the square four‐point probe method^[^ ^44^ ^]^ (KEITHLEY instrument, 2450 SourceMeter). The electrical resistivity (ρ) was calculated through the equation *R_s_* =  C · ρ/*t*, where *t* is the thickness of the Cu NM and C is the correction factor (refer to ref. [45]). Additionally, C‐AFM experiments were conducted on the Cu NMs transferred onto glass slices using conductive diamond probes (Adama Innovations, NC‐LC) under an applied voltage of 5 mV.

### Thermal Property Characterization of Cu NMs

To assess the thermal properties of the Cu NMs, F‐DSC experiments were conducted using the Mettler Flash‐DSC 2+ and isothermal annealing experiments using the Linkam TS1400XY heating stage. The F‐DSC experiments were performed with the temperature ranging from −70 to 1000 °C and the heating rate varying from 100 to 10 000 K s^−1^. Additionally, isothermal annealing experiments of these Cu NMs on TEM grids were performed under an Argon atmosphere annealing at 573 or 673 K for a duration of 30 s. The grain growth in the annealed Cu NMs was observed using conventional field emission TEM.

### Mechanical Characterization of Cu NMs and Substrates

To assess the mechanical properties of Cu NMs, AFM indentation was employed following the widely used circular drum indentation method.^[^ ^28^ ^]^ The freestanding Cu NMs were transferred onto patterned Si wafers with a series of circular holes (diameter of 3 µm). AFM indentation tests were then conducted on the suspended Cu NMs with diamond probes (Adama Innovations, NC‐LC). As described in previous studies,^[^ ^29^, ^30^ ^]^ the elastic modulus, yielding strength, and ductility of the Cu NMs were derived by fitting the experimental data to finite element analysis (FEA) using the commercial software ANSYS (ANSYS Inc., USA). An axisymmetric finite element model was constructed for the AFM indentation tests based on the experimental conditions, including the hole radius, tip radius, NM thickness, and profile. For simplicity, a Poisson's ratio of 0.35 was assumed based on the property of bulk Cu. By utilizing elastic and elastoplastic constitutive equations, the elastic modulus, and the yield strength of the Cu NMs was obtained by fitting the experimental force‐displacement curves. The maximum von Mises strain before strain softening was determined as the ductility of the Cu NMs.

The elastic modulus of the Cu substrate was determined through nanoindentation using a Berkovich tip on the Bruker TI950 nanoindentation system. We applied partial load‐unload functions with 20 cycles over a period of 60 s, with a maximum load of 4 mN. To ensure data reliability, the indentation tests were repeated three times on different samples. The elastic modulus of the Cu substrate was determined by fitting the measured reduced modulus as a function of indentation depth to the King's model.^[^ ^36^ ^]^

### Cu─Cu Bonding Experiments and Shear Tests

All bonding experiments were conducted using the bonding machine (ETOOL, ET‐1212‐300) without any surface treatment, applying a minimum force of 100 N to ensure contact between the two Cu surfaces to be bonded. For Cu─Cu direct bonding (Figure S11a, Supporting Information), the optimal bonding parameters were determined by performing bonding experiments at various bonding temperatures (100, 200, and 300 °C) and time periods (3, 30, and 300 min), as illustrated in Figure S12 (Supporting Information). In the case of Cu NM enabled Cu─Cu bonding, a selected number of Cu NMs were transferred onto the surface of Cu substrates (Video S2, Supporting Information), ensuring that the Cu NMs were sandwiched between the Cu substrates to be bonded. Cu NM enabled Cu─Cu bonding (Figure S11b, Supporting Information) was carried out at 300 °C with annealing times of 3, 30, or 300 min.

Following the approach described in refs. [37, 38], shear tests were performed on the bonded Cu─Cu surfaces using the Nordson Dage 4000 Bond Tester. The tests were conducted both with and without Cu NMs, as shown in Videos S3 and S4 (Supporting Information). The shear height and rate were maintained at 50 and 100 µm s^−1^, respectively. Shear stress (τ) was calculated using the formula τ  =  *F*/*A*, where *F* represents the shear force, and *A* is the nominal bonding area, which is equal to the area of the Cu substrate deposited on the Si wafer.

### Characterization of Bonded Interface

The bonded Cu─Cu interface was examined using conventional field emission TEM and Cs‐corrected thermal field emission TEM. To prepare the TEM samples of the bonded interface, the FEI SEM/focused‐ion beam (SEM/FIB) system was utilized. Initially, a 2 µm‐thick Pt protection layer was deposited on the bonded interface region. Subsequently, a rectangular lamina was created measuring 10 µm × 7 µm × 1.4 µm from this region and transferred it on a Mo grid. The lamina's thickness was reduced to less than 100 nm from both sides using the gallium ion beam at a tilt angle of ±1.2° and an accelerating voltage of 30 kV. The lamina sequentially was then polished using an ion beam of 48 pA at 5 kVand of 27 pA at 2 kV.

### Molecular Dynamics (MD) Simulations

MD simulations of copper (Cu) and cuprous oxide (Cu_2_O) were performed using the third‐generation charge‐optimized many body potential (COMB3),^[^ ^46^ ^]^ implemented in LAMMPS.^[^ ^47^ ^]^ Bi‐crystal Cu models with different GB misorientation tilt angles (Σ5[001](210) and Σ41[001](910)), as well as Cu─Cu_2_O─Cu models, were generated using Atomsk.^[^ ^48^ ^]^ The Cu models had dimensions of 7.23 × 7.23 × 14.46 nm^3^, while the Cu─Cu_2_O─Cu models had dimensions of 7.23 × 7.23 × 18.075 nm^3^. The models were annealed at 573 K for 100 ps, followed by cooling to 300 K in 1 ps and equilibration for 20 ps under the NVT ensemble. A timestep of 0.1 fs was used, and periodic boundary conditions were applied in all three directions. Electronegativity equilibration was performed every 10 timesteps during the simulations. To calculate the thermodynamic driving forces for bonding, the corresponding unbonded models with free surfaces were simulated using the same procedure. The energy differences per unit surface area between the two models, Δ*E*, were evaluated at 573 K.

Uniaxial tension and simple shear simulations were conducted on the annealed bonded models at a nominal strain rate of 5 × 10^9^ s^−1^, still under the NVT ensemble. To investigate the effect of oxide content on the mechanical properties, uniaxial tension was performed on Cu_2_O particle‐embedded copper models with varying particle radius. These models had dimensions of 7.23 × 7.23 × 7.23 nm^3^.

## Conflict of Interest

Y.Y., Y.T., W.Z., and H.G. are in the process of applying a patent related to the Cu‐Cu nanomembrane bonding described in this work. The remaining authors declare no competing interests.

## Supporting information



Supporting Information

Supplemental Video 1

Supplemental Video 2

Supplemental Video 3

Supplemental Video 4

## Data Availability

The data that support the findings of this study are available from the corresponding author upon reasonable request.
