# Supplementary material for: Ultra‐Tough Copper–Copper Bonding by Nano‐Oxide‐Dispersed Copper Nanomembranes
Source: Adv Sci (Weinh). 2025 Feb 14;12(14):2408302. doi: 10.1002/advs.202408302 (PMC11984842; doi:10.1002/advs.202408302)
Supplement: Supplementary file 1 — Supporting Information [file ADVS-12-2408302-s002.pdf]

ADVANCED SCIENCE
Open Access

Supporting Information

for *Adv. Sci.*, DOI 10.1002/advs.202408302

Ultra-Tough Copper–Copper Bonding by Nano-Oxide-Dispersed Copper Nanomembranes

*Yun Teng, Wenqing Zhu, Qing Wang, Zhibo Zhang, Hang Wang, Baisong Guo, Ziyin Yang, Hao Gong, Chuan He, Boxi Qu, Shien-Ping Feng and Yong Yang**

Supplementary Materials

Ultra-Tough Copper-Copper Bonding by Nano-Oxide-Dispersed Copper Nanomembranes

Yun Teng[1, †], Wenqing Zhu[1,2, †], Qing Wang[3], Zhibo Zhang[1], Hang Wang[1], Baisong Guo[4], Ziyin Yang[1], Hao Gong[1], Chuan He[5], Boxi Qu[6], Shien-Ping Feng[5], Yong Yang[1,5,7, *]

1. Department of Mechanical Engineering, City University of Hong Kong, Tat Chee Avenue, Kowloon Tong, Kowloon, Hong Kong, China

2. State Key Laboratory for Turbulence and Complex System, Department of Mechanics and Engineering Science, College of Engineering, Peking University, Beijing, China.

3. Laboratory for Microstructures, Institute of Materials, Shanghai University, Shanghai, China

4. Institute of Advanced Wear & Corrosion Resistant and Functional Materials, Jinan University, Guangzhou, Guangdong, China

5. Department of Systems Engineering, City University of Hong Kong, Tat Chee Avenue, Kowloon Tong, Kowloon, Hong Kong, China

6. Nano and Advanced Materials Institute, Science Park West Avenue, Hong Kong Science Park, Hong Kong, China

7. Department of Materials Science and Engineering, City University of Hong Kong, Tat Chee Avenue, Kowloon Tong, Kowloon, Hong Kong, China

† Y.T. and W.Z. contributed equally to this work

* Corresponding author. Email: yonyang@cityu.edu.hk

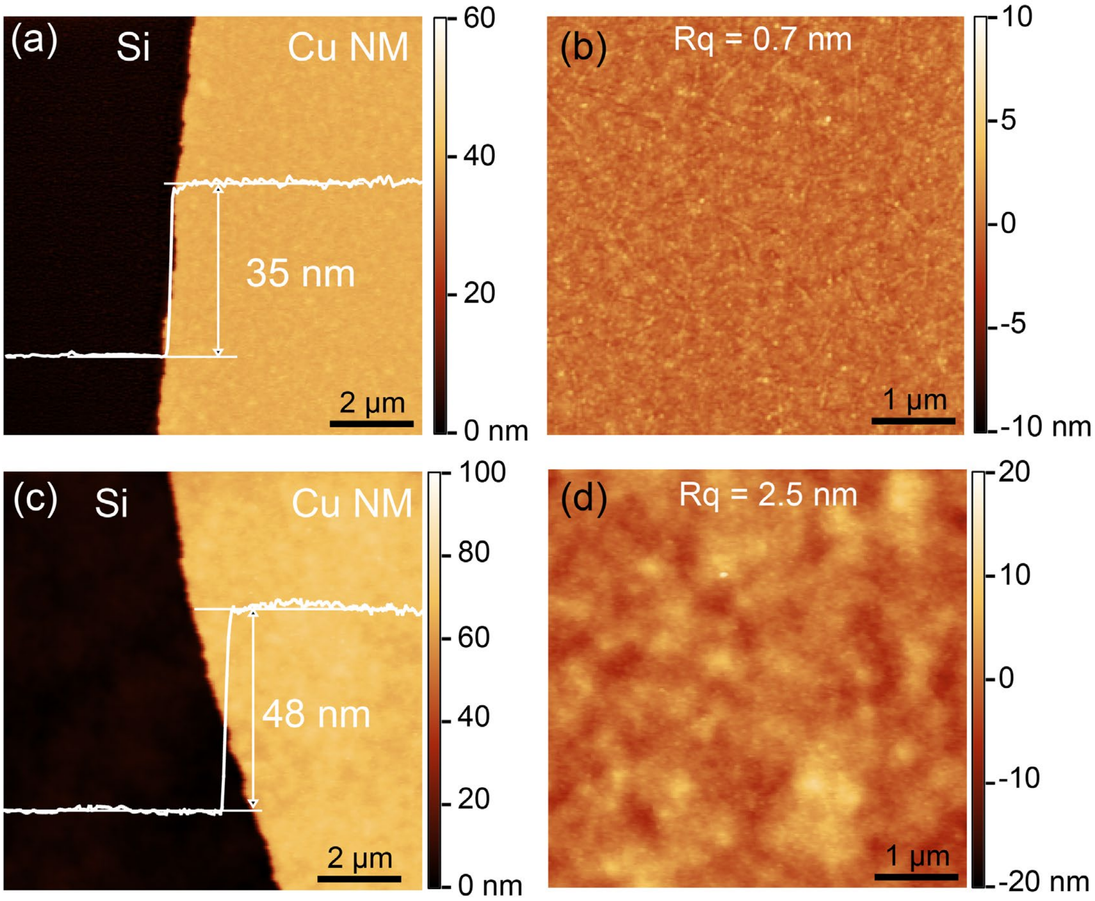

Figure S1. (a) Atomic force microscope (AFM) topographic image of the 35 nm-thick Cu nanomembrane (NM) on silicon. The insert is the line scan profile across the edge of the 35 nm-thick Cu NM. **(b)** Root mean square roughness (Rq) of the 35 nm-thick Cu NM. **(c)** AFM topographic image of the 48 nm-thick Cu NM on silicon. The insert is the line scan profile across the edge of the 48 nm-thick Cu NM. **(f)** Rq of the 48 nm-thick Cu NM.

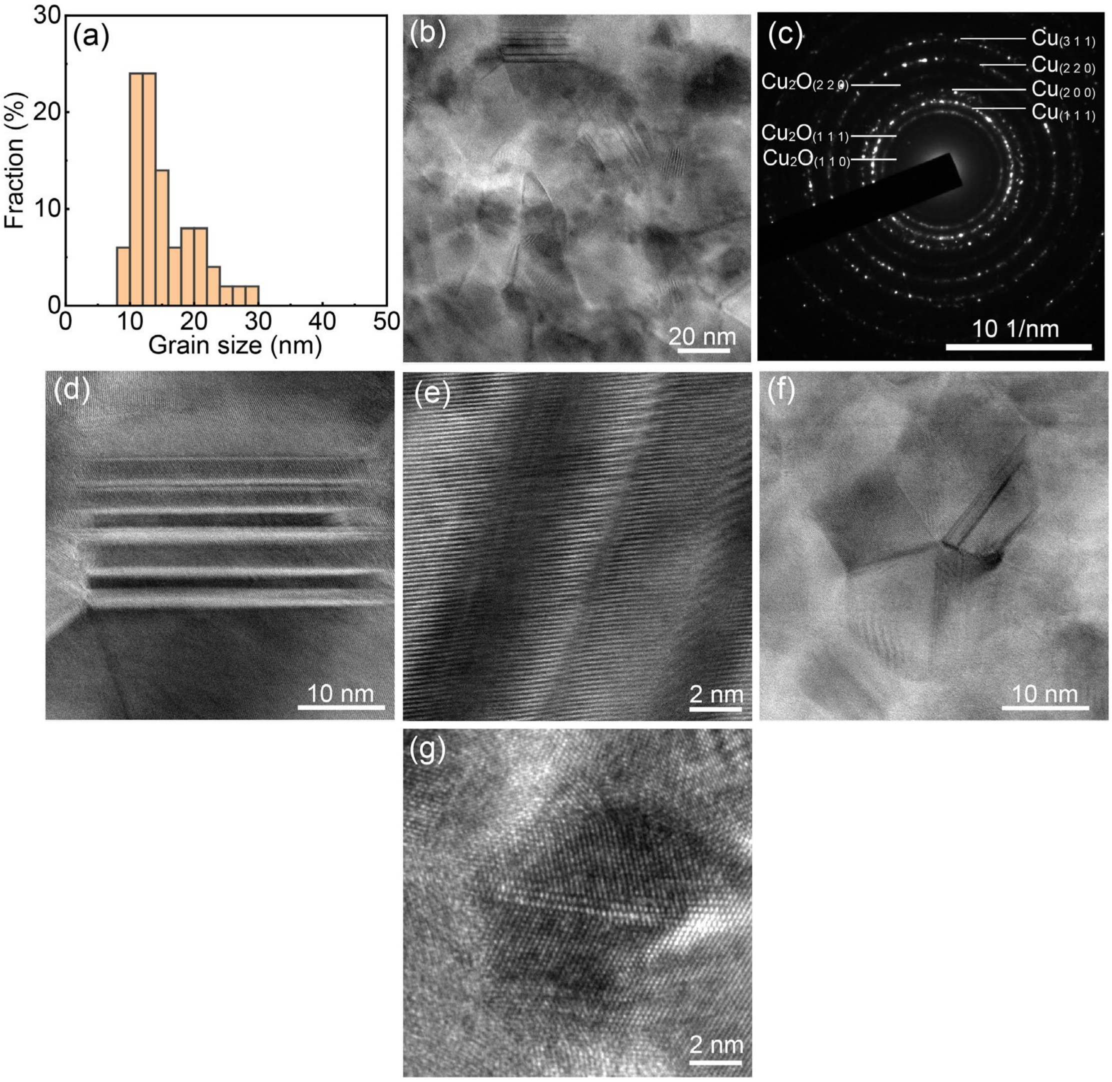

Figure S2. (a) Grain size distribution in Cu NM with an average grain size of 16 nm. **(b)** Transmission electron microscopy (TEM) image of a typical 48 nm-thick Cu NM. **(c)** The selective area electron diffraction (SAED) pattern of the 48 nm-thick Cu NM. The defects in Cu NM: **(d-e)** stacking fault and **(f-g)** twin boundary.

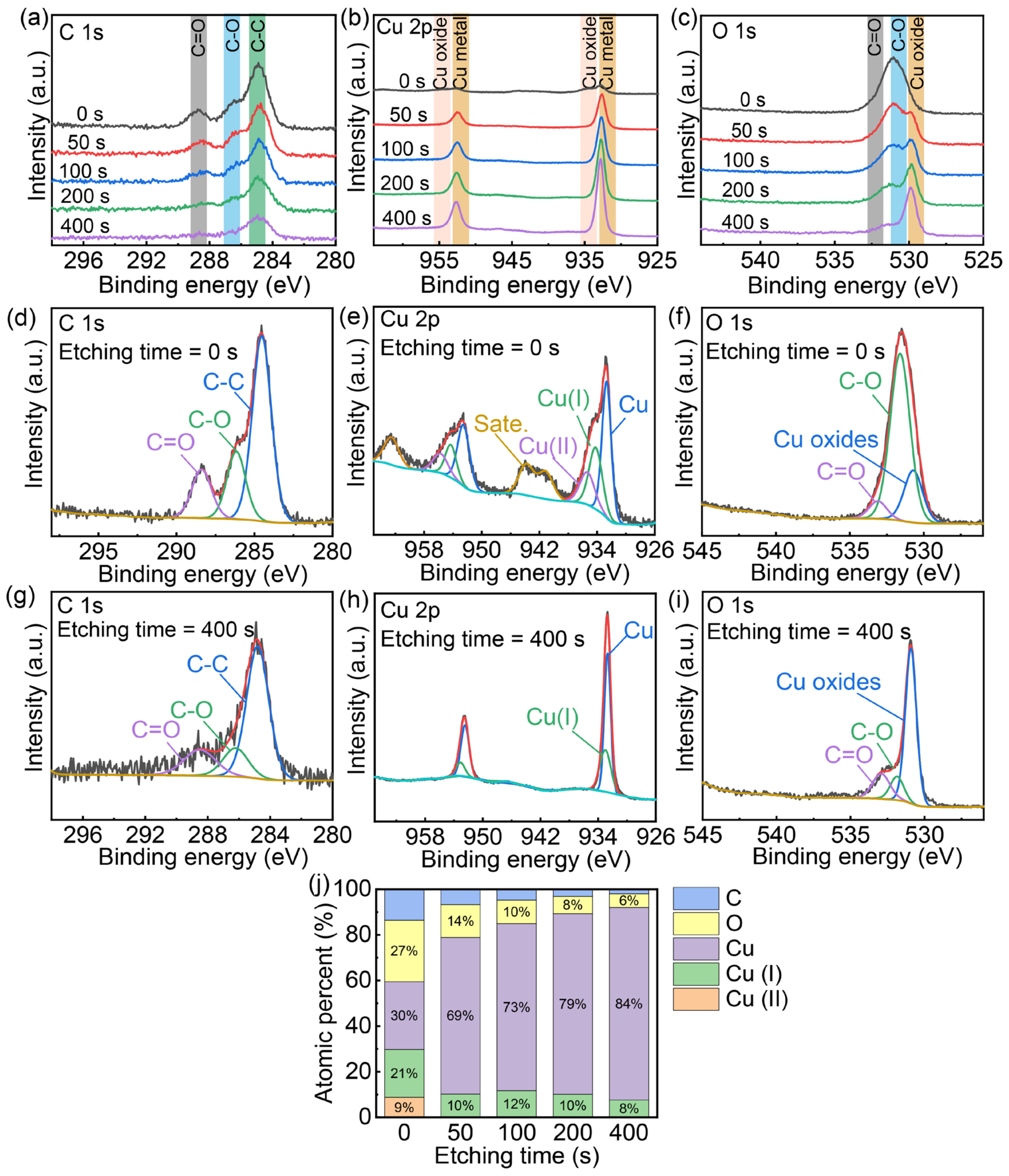

Figure S3. XPS depth profile analysis of the 48 nm-thick Cu NM. Narrow-scan XPS spectra of **(a)** C 1s, **(b)** Cu 2p, and **(c)** O 1s as a function of etching time. The XPS spectrum of narrow-scan XPS spectra for **(d)** C 1s, **(e)** Cu 2p and **(f)** O 1s with etching time of 0 s. The XPS spectrum of narrow-scan XPS spectra for **(g)** C 1s, **(h)** Cu 2p and **(i)** O 1s at etching time of 400 s. **(j)** Atomic percentage of Cu, C and O with different etching time.

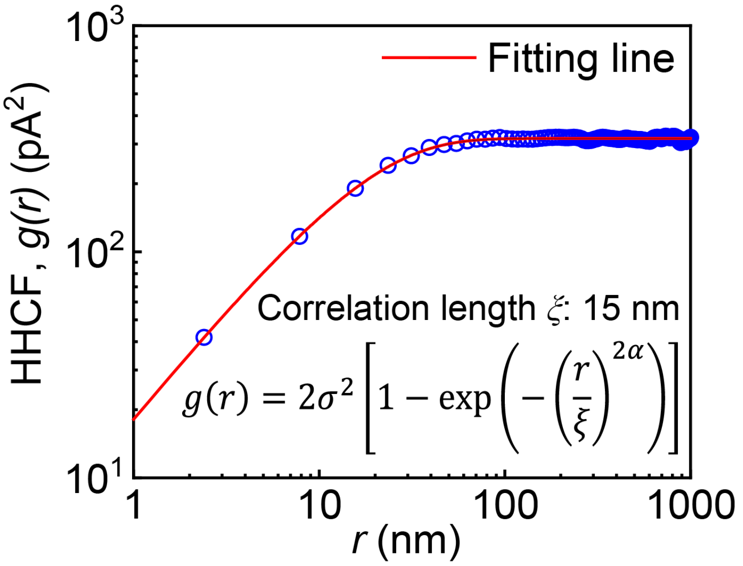

Figure S4. The calculated height-height correlation function (HHCF) from **Figure 1(h)**.

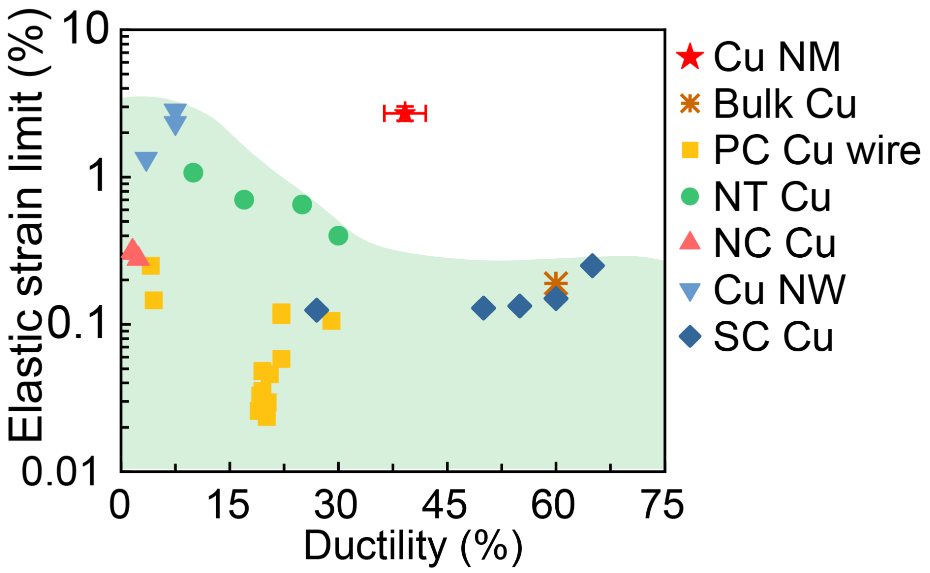

Figure S5. Comparison of the elastic strain limit and ductility of Cu NM with those of bulk Cu[1], polycrystalline (PC) Cu wires[2], nanocrystalline (NC) Cu[3], nanotwinned (NT) Cu[1], Cu nanowires (NWs) [4] and single crystalline (SC) Cu[5].

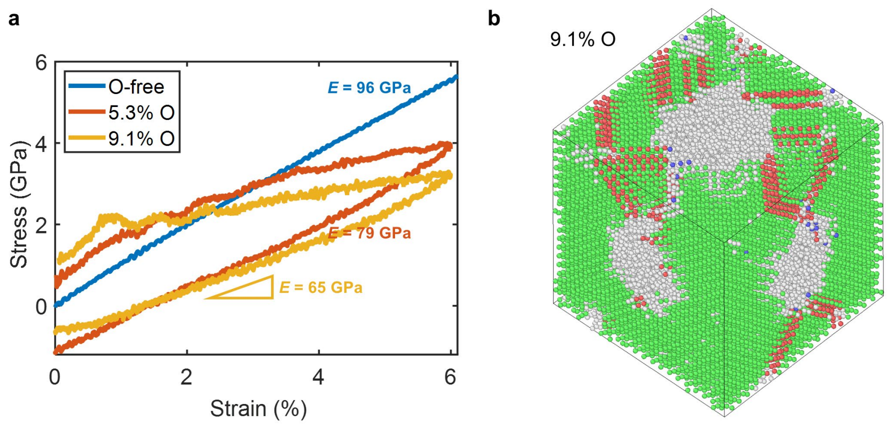

Figure S6. **(a)** Stress-strain curves of pure Cu, Cu with 5.3% O and 9.1% O. **(b)** Atomic structure of Cu with 9.1% O at the unloading stage showing stacking-fault defects. Green, red and blue spheres represent FCC, HCP and BCC atoms, respectively while gray color represents other types.

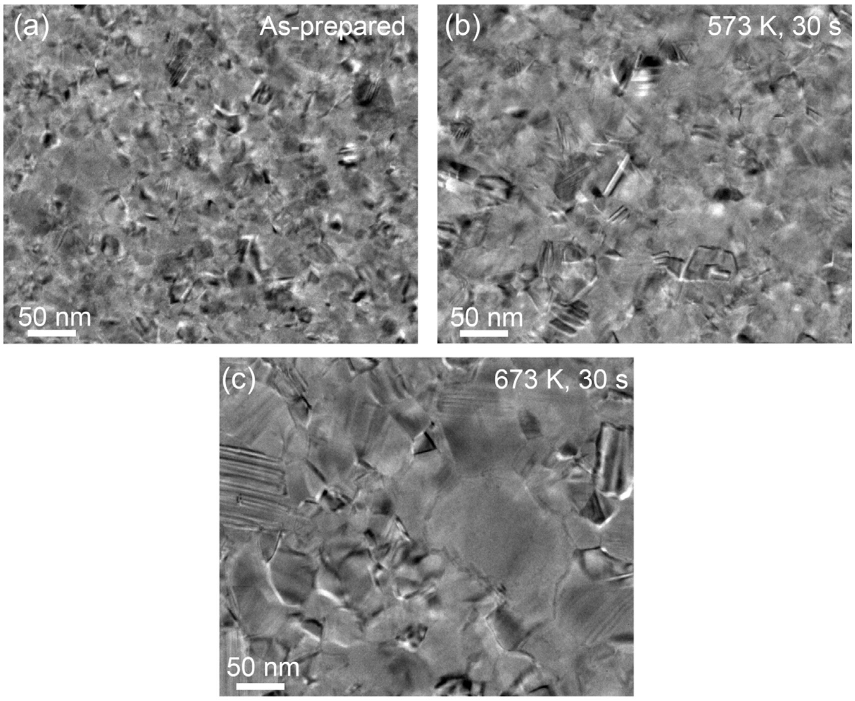

Figure S7. TEM images of **(a)** 48 nm-thick as-prepared Cu NM and those NMs after iso-thermal annealing for 30s at **(b)** 573 K and **(c)** 673 K.

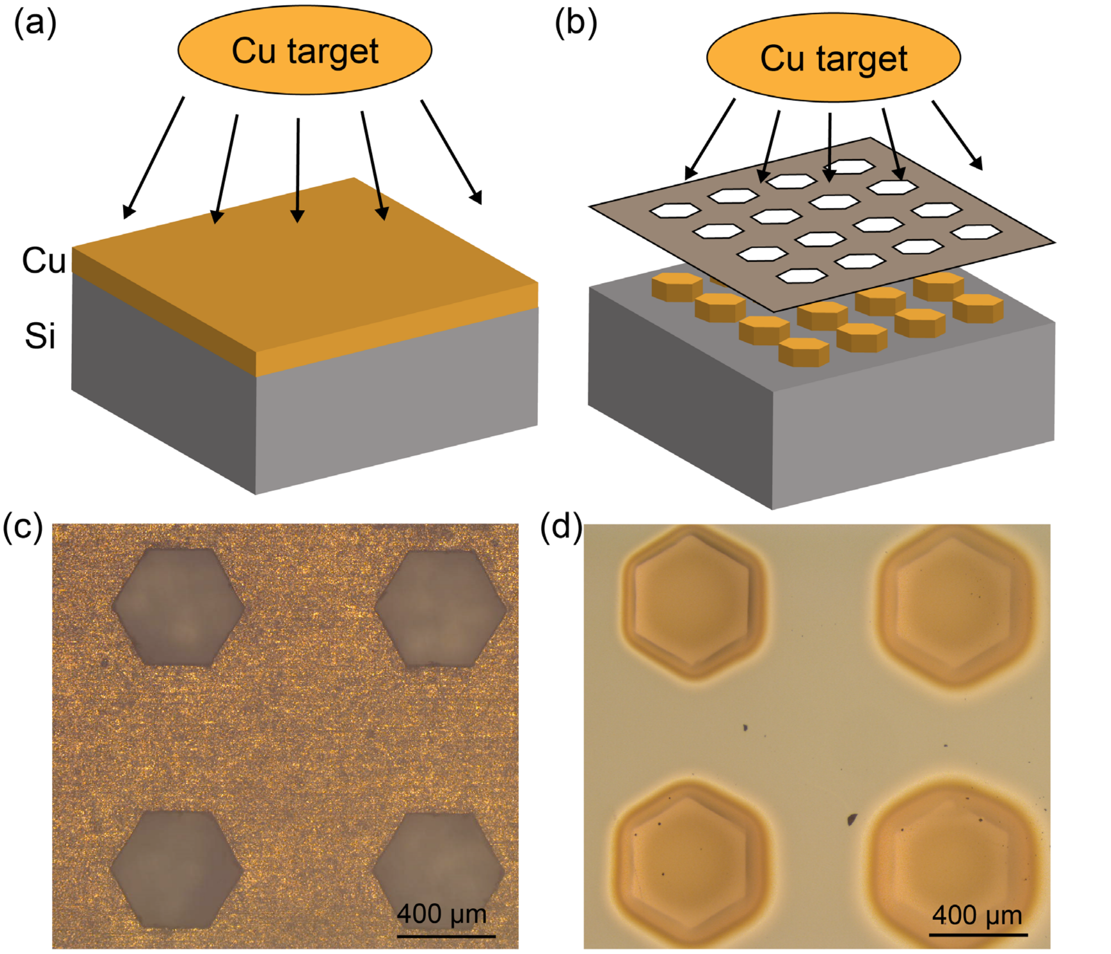

Figure S8. Illustration of the deposition process of **(a)** unpatterned and **(b)** patterned Cu substrate. Optical images of **(c)** the deposition mask and **(d)** deposited patterned Cu substrate.

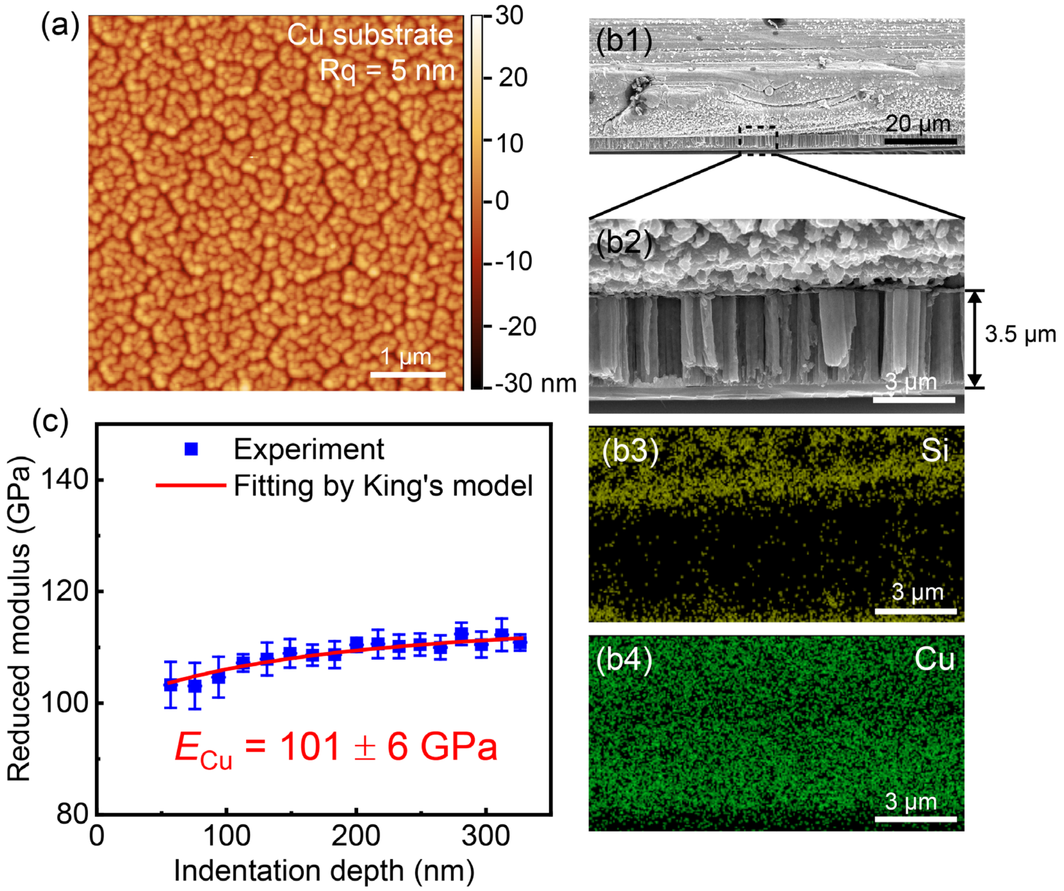

Figure S9. **(a)** Rq of Cu substrate deposited on Si wafer. **(b1-b2)** Side views of 3.5 μm Cu substrate deposited on Si wafer by SEM. **(b3-b4)** Energy Dispersive Spectroscopy (EDS) mapping of **(b2)**. **(c)** Elastic modulus of Cu substrate obtained from nanoindentation tests and fitting by King's model.[6]

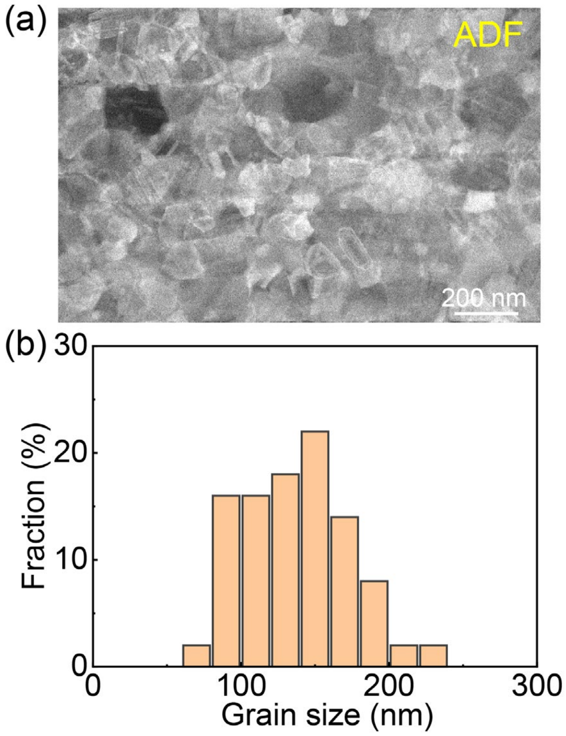

Figure S10. (a) Annular dark-field (ADF) TEM images of Cu substrate deposited on Silicon. **(b)** Grain size distribution in Cu substrate with an average grain size of 136 nm.

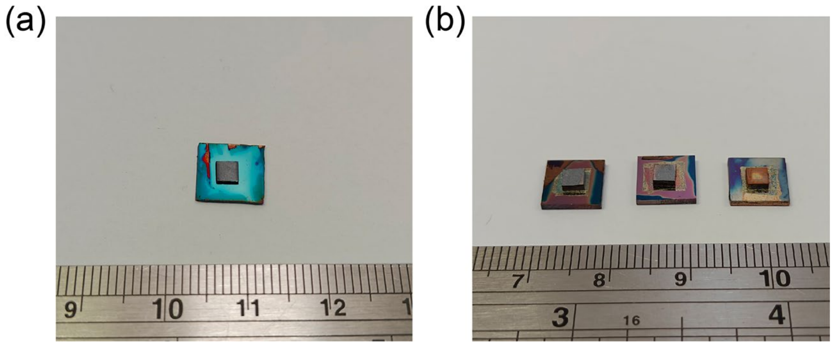

Figure S11. (a) Optical image of direct Cu-Cu bonding sample without Cu NM. **(b)** Optical image of Cu-Cu bonding samples with Cu NMs.

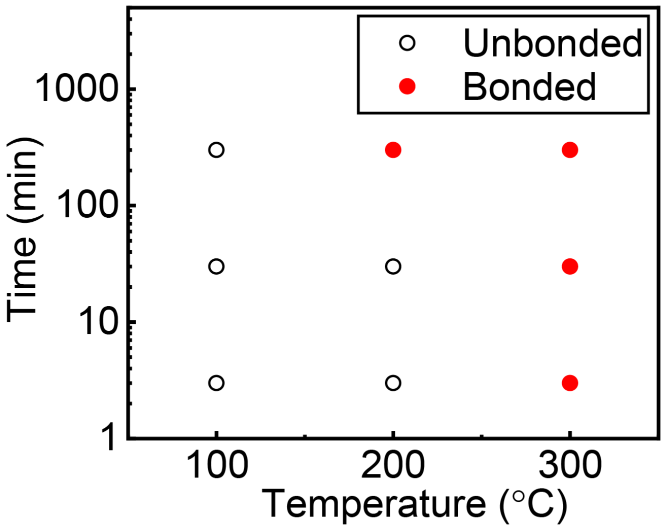

Figure S12. Bonding results of direct Cu-Cu bonding experiments without Cu NMs.

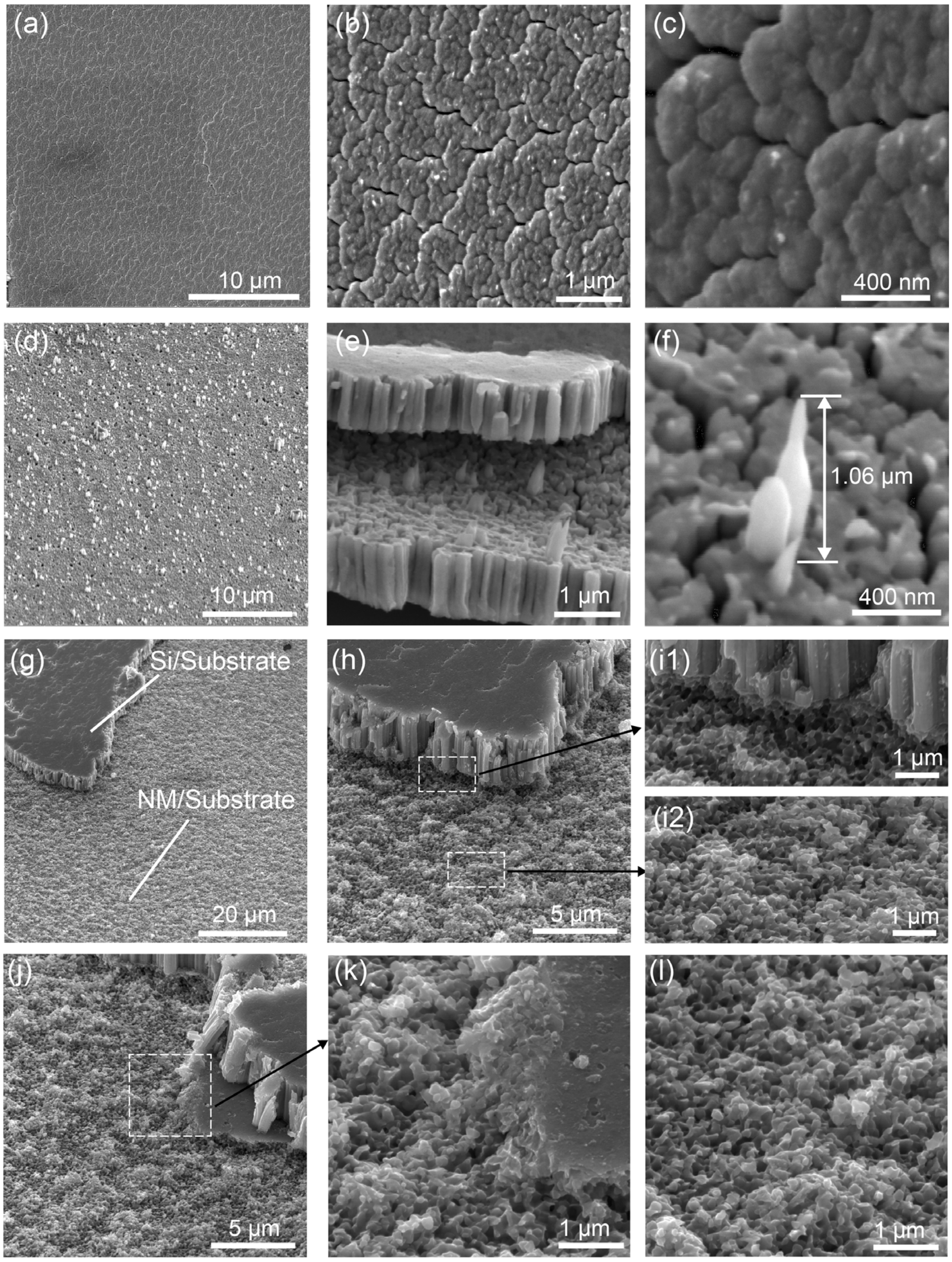

Figure S13. Fractured surface of Cu-Cu bonding samples. **(a-c)** SEM images of fractured surface of Cu-Cu bonding sample without Cu NM. **(d-f)** SEM images of fractured surface of Cu-Cu bonding sample with one-layer Cu NM. **(g-i)** SEM images of fractured surface on the one side of Cu-Cu bonding sample with three-layer Cu NMs. **(j-l)** SEM images of fractured surface on the other side of Cu-Cu bonding sample with three-layer Cu NMs. All SEM images were taken at a tilt angle of 45° with respect to normal incidence.

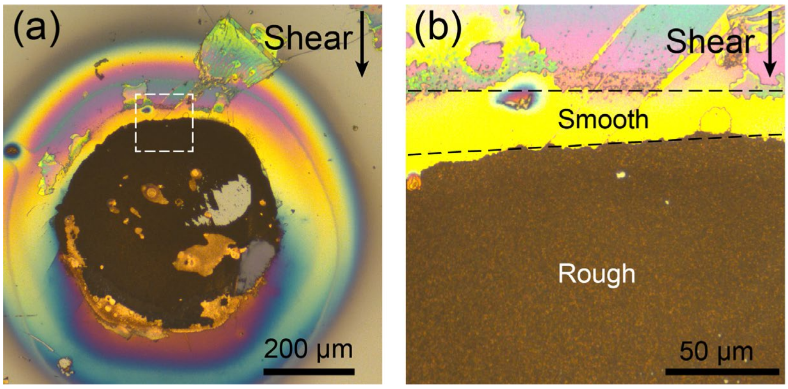

Figure S14. (a) Optical images of the fracture surface of Cu-Cu NM bonding with three-layer Cu NMs. **(b)** Magnified optical images of the fracture surface of Cu-Cu NM bonding with three-layer Cu NMs in **(a)**. The black solid arrows in **(a)** and **(b)** indicate the shear direction.

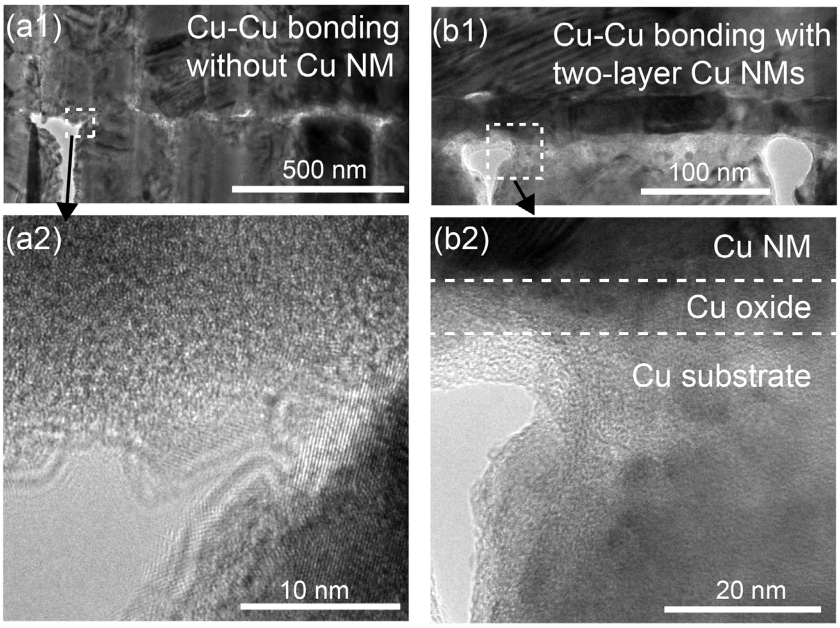

Figure S15. (a1) TEM images of the bonding interface of Cu-Cu direct bonding without Cu NM. **(a2)** Magnified TEM images of the crack tip for Cu-Cu direct bonding in **(a1)**. **(b1)** TEM images of the bonding interface of Cu-Cu NM bonding with two-layer Cu NMs. **(b2)** Magnified TEM images of the blunt crack tip for Cu-Cu NM bonding in **(b1)**.

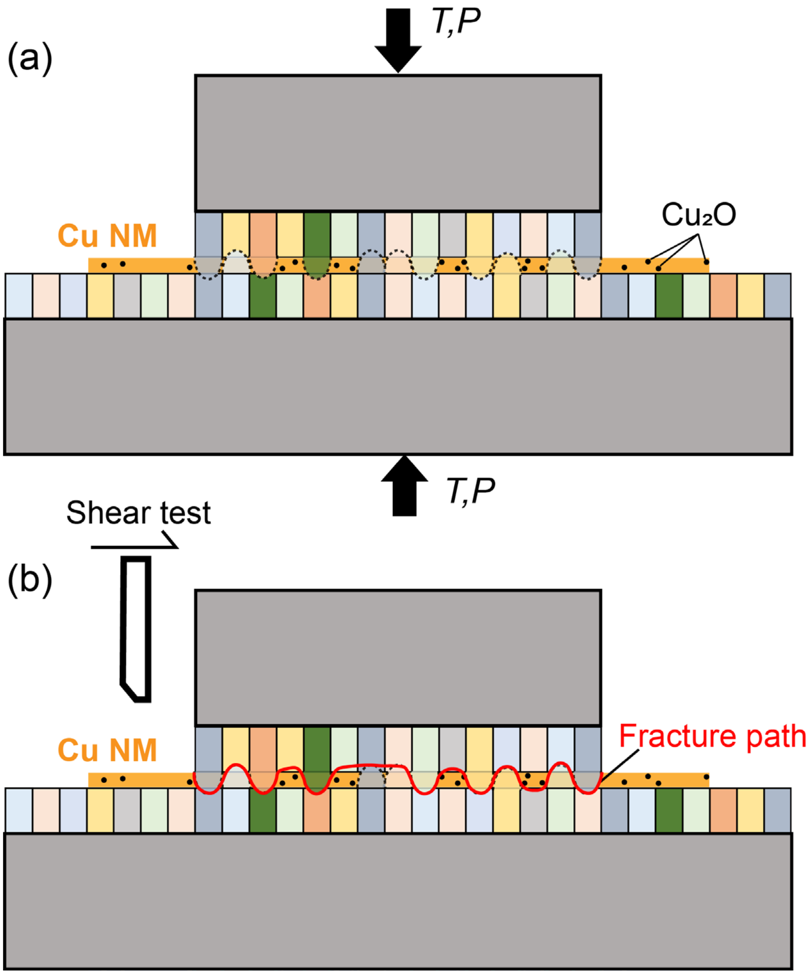

Figure S16. (a) Illustration of the grain growth in Cu-Cu NM bonding. The dashed lines indicate the grain growth front. The black points represent the Cu_2O nano-oxides. **(b)** Illustration of the tortuous fracture process resulting from Cu-Cu NM bonding.

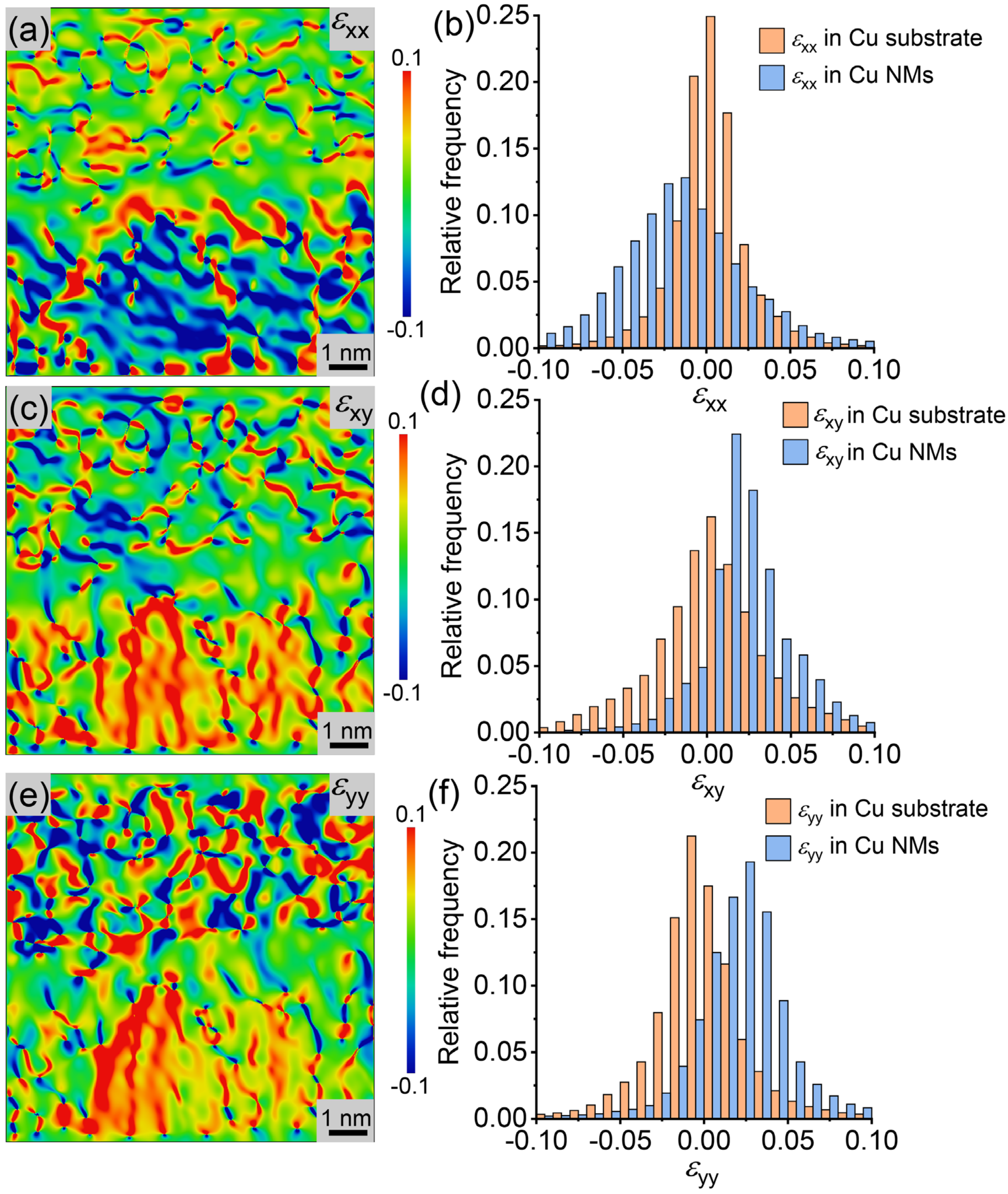

Figure S17. (a) Contour plot and **(b)** distribution of the normal strain ε_{xx} in **Figure 5(c)**. **(c)** Contour plot and **(d)** distribution of the shear strain ε_{xy} in **Figure 5(c)**. **(e)** Contour plot and **(f)** distribution of the normal strain ε_{yy} in **Figure 5(c)**.

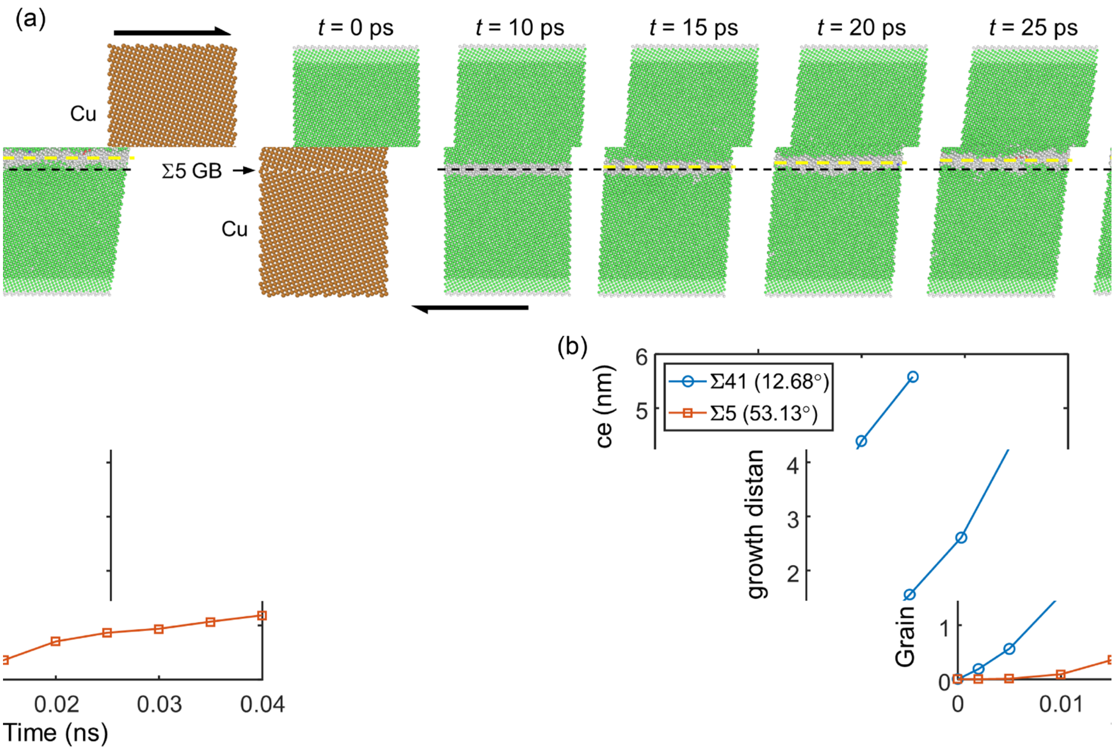

Figure S18. Misorientation-dependent grain boundary migration kinetics. (a) Snapshots of the bi-crystal copper model with Σ5[001](210) GB subject to simple shear. (b) Grain growth distance versus time for Σ5[001](210) and Σ41[001](910) bi-crystal models.

Table S1. Comparison of the electrical resistivity of Cu NM with those of CuO, Cu_2O, NC and NT Cu, and bulk Cu.

Materials	Resistivity (μΩ·cm)	References
CuO	4.8×10^{10}	[7]
Cu_2O	1.6×10^{10}	[7]
**Cu NM**	**25 ± 19**	This work
NC Cu	20	[1]
NT Cu	2.0	[1]
Bulk Cu	1.6	[1]

Table S2. Comparison of environmental friendliness and cost of the various Cu-Cu bonding techniques.

Bonding techniques	Strength (MPa)	Procedures	Environmental friendliness	Cost	References
Cu-Cu nanoparticle bonding	6-48	**Production of nanoparticle:** need aqueous NaOH and nitrilotriacetic acid disodium salt, hydrazine monohydrate (toxic), oleylamine.	No	High	[8]
		**Deoxidization of nanoparticles:** need oxalic acid or formic acid or phosphoric acid.	No		
		**Surface treatment:** need sulfuric acid.	No		
		**Bonding process:** bonding under Ar atmosphere under pressure of 0 MPa to 10 MPa from 175 to 300 °C for 5 to 60 min.	Yes		
Cu-Cu nanowire bonding	13-60	**Production of nanowire:** need NaOH	No	High	[9]
		**Deoxidization of nanowires:** cleaned with citric acid.	Yes		
		**Surface treatment:** cleaned in an oxygen plasma.	Yes		
		**Bonding process:** bonded in nitrogen atmosphere at 230 °C for 120 s under pressure of 1 to 10 MPa.	Yes		
Direct Cu-Cu bonding	2-57	**Production of (111) oriented Cu substrate:** Magnetron sputtering or electroplating, need acetic acid, phosphoric acid, HCl.	No	High	[10]
		**Surface treatment:** need phosphoric acid or HCl or chemical mechanical planarization (CMP).	No		
		**Bonding process:** bonded at 200°C ~ 300 °C for 10 min ~ 90 min under pressure of 1 to 22 MPa in vacuum or nitrogen atmosphere.	Yes		
Cu-Cu bonding with metal passivation layer	13-28	**Production of metal passivation layer:** deposited by PVD deposition.	Yes	Medium	[11]
		**Surface treatment:** plasma treatment.	Yes		
		**Bonding process:** bonded at 120°C ~ 200 °C for 3 min ~ 50 min under pressure of 1.27 MPa in vacuum or nitrogen atmosphere.	Yes		
Cu-Cu bonding with Cu NMs	4-73	**Production of Cu nanomembranes:** deposited by PVD deposition and peeled off in DI water, without surface treatment	Yes	Low	This work

	**Bonding process:** bonded in nitrogen atmosphere at 300°C for 3 min ~ 300 min under pressure less than 10 MPa.	Yes

Supplementary References

[1] L. Lu, Y. Shen, X. Chen, L. Qian, K. Lu, *Science* **2004**, *304*, 422.

[2] a) B. Yang, C. Motz, M. Rester, G. Dehm, *Philos. Mag.* **2012**, *92*, 3243; b) Y. Q. Zhang, S. Y. Jiang, *T Nonferr Metal Soc* **2021**, *31*, 1381.

[3] a) P. G. Sanders, J. A. Eastman, J. R. Weertman, *Acta Mater.* **1997**, *45*, 4019; b) P. Chen, Z. Zhang, C. Liu, T. An, H. Yu, F. Qin, *Modell. Simul. Mater. Sci. Eng.* **2019**, *27*.

[4] a) H. A. Wu, A. K. Soh, X. X. Wang, Z. H. Sun, *Key Eng. Mater.* **2004**, *261-263*, 33; b) W. Liang, M. Zhou, *P I Mech Eng C-J Mec* **2004**, *218*, 599; c) A. R. Alian, Y. Ju, S. A. Meguid, *Mater. Des.* **2019**, *175*; d) S. Tardieu, D. Mesguich, A. Lonjon, F. Lecouturier, N. Ferreira, G. Chevallier, A. Proietti, C. Estournès, C. Laurent, *Mater. Sci. Eng. A* **2019**, *761*.

[5] T. Guo, S. Wei, C. Wang, Q. Li, Z. Jia, *Mater. Sci. Eng. A* **2019**, *759*, 97.

[6] R. B. King, *Int. J. Solids Struct.* **1987**, *23*, 1657.

[7] L. De Los Santos Valladares, D. H. Salinas, A. B. Dominguez, D. A. Najarro, S. I. Khondaker, T. Mitrelias, C. H. W. Barnes, J. A. Aguiar, Y. Majima, *Thin Solid Films* **2012**, *520*, 6368.

[8] a) D. Namgoong, Y. Kim, K. Siow, J. H. Lee, *Available at SSRN 4341143* **2023**; b) Y. Kamikoriyama, H. Imamura, A. Muramatsu, K. Kanie, *Sci. Rep.* **2019**, *9*, 899; c) Y. Gao, H. Zhang, W. Li, J. Jiu, S. Nagao, T. Sugahara, K. Suganuma, *J. Electron. Mater.* **2017**, *46*, 4575; d) Y. Gao, W. Li, C. Chen, H. Zhang, J. Jiu, C. F. Li, S. Nagao, K. Suganuma, *Materials & Design* **2018**, *160*, 1265; e) I. Kim, Y. Kim, K. Woo, E. H. Ryu, K. Y. Yon, G. Cao, J. Moon, *RSC Adv.* **2013**, *3*, 15169; f) Y. Kobayashi, T. Shirochi, Y. Yasuda, T. Morita, *Int. J. Adhes. Adhes.* **2012**, *33*, 50; g) Y. Mou, Y. Peng, Y. Zhang, H. Cheng, M. Chen, *Mater. Lett.* **2018**, *227*, 179; h) T. Ishizaki, R. Watanabe, *J. Mater. Chem.* **2012**, *22*, 25198; i) Y. Zuo, J. Shen, J. Xie, L. Xiang, *J. Mater. Process. Technol.* **2018**, *257*, 250; j) T. Yamakawa, T. Takemoto, M. Shimoda, H. Nishikawa, K. Shiokawa, N. Terada, *J. Electron. Mater.* **2013**, *42*, 1260; k) X. Liu, H. Nishikawa, *Scr. Mater.* **2016**, *120*, 80.

[9] a) F. Roustaie, S. Quednau, F. Dassinger, O. Birlem, in *2020 15th International Microsystems, Packaging, Assembly and Circuits Technology Conference (IMPACT)* IEEE, **2020**, 168-171; b) F. Roustaie, S. Quednau, F. Weissenborn, O. Birlem, D. Riehl, X. Ding, A. Kramer, K. Hofmann, in *2021 IEEE 71st Electronic Components and Technology Conference (ECTC)* IEEE, **2021**, 371-376; c) D. Strahringer, F. Roustaie, F. Weissenborn, S. Quednau, J. Wilde, in *2021 16th International Microsystems, Packaging, Assembly and Circuits Technology Conference (IMPACT)* IEEE, **2021**, 31-34; d) Z. Yu, Y. Z. Tan, C. F. Bayer, H. Rauh, A. Schletz, M. März, O. Birlem, in *2021 IEEE 23rd Electronics Packaging Technology Conference (EPTC)* IEEE, **2021**, 1-7.

[10] a) C. M. Liu, H. W. Lin, Y. S. Huang, Y. C. Chu, C. Chen, D. R. Lyu, K. N. Chen, K. N. Tu, *Sci. Rep.* **2015**, *5*, 9734; b) C. N. Li, W. L. Chiu, H. H. Chang, C. Chen, in *2024 International Conference on Electronics Packaging (ICEP)* **2024**, 109-110; c) T. Sakai, N. Imaizumi, T. Miyajima, in *2012 2nd IEEE CPMT Symposium Japan* IEEE, **2012**, 1-4; d) K. C. Shie, J. Y. Juang, C. Chen, *Japanese Journal of Applied Physics* **2019**, *59*, SBBA03.

[11] a) D. Liu, P. C. Chen, T. C. Chou, H. W. Hu, K. N. Chen, *IEEE J. Electron Devices Soc.* **2021**, *9*, 868; b) Z. J. Hong, D. Liu, H. W. Hu, M. C. Lin, T. H. Hsieh, K. N. Chen, in *2021 IEEE 71st Electronic Components and Technology Conference (ECTC)* IEEE, **2021**, 347-352; c) T. C. Chou, S. Y. Huang, P. J.

Chen, H. W. Hu, D. Liu, C. W. Chang, T. H. Ni, C. J. Chen, Y. M. Lin, T. C. Chang, *IEEE Trans. Compon. Packag. Manuf. Technol.* **2020**, *11*, 36.
